# A novel ubiquitin-related genes-based signature demonstrated values in prognostic prediction, immune landscape sculpture and therapeutic options in laryngeal cancer

**DOI:** 10.3389/fphar.2025.1513948

**Published:** 2025-03-20

**Authors:** Lu Liu, Bing Wang, Xiaoya Ma, Lei Tan, Xudong Wei

**Affiliations:** ^1^ The First Clinical Medical College of Lanzhou University, Lanzhou, China; ^2^ Department of E.N.T., Gansu Provincial Hospital, Lanzhou, China; ^3^ Center for Energy Metabolism and Reproduction, Institute of Biomedicine and Biotechnology, Shenzhen Institute of Advanced Technology, Chinese Academy of Sciences, Shenzhen, China; ^4^ Innovation Center of Suzhou Nanjing Medical University, Suzhou, China; ^5^ Pediatric Heart Disease Treatment Center, Jiangxi Provincial Children’s Hospital, Nanchang, China; ^6^ Department of Cardiology, Shenzhen Guangming District People’s Hospital, Shenzhen, China; ^7^ State Key Laboratory of Reproductive Medicine and Offspring Health, Nanjing Medical University, Nanjing, China; ^8^ National Center of Technology Innovation for Biopharmaceuticals, Suzhou, China

**Keywords:** laryngeal cancer, ubiquitin-related genes-based signature, prognosis prediction, immune landscape sculpture, therapeutic options

## Abstract

**Background:**

Laryngeal cancer (LC) is characterized by high mortality and remains challenging in prognostic evaluation and treatment benefits. Ubiquitin-related genes (UbRGs) are widely involved in cancer initiation and progression, but their potential value in LC is unknown.

**Methods:**

RNA-seq and clinical data of LC were obtained from TCGA and GEO. UbRGs that independently influenced the overall survival (OS) of LC patients were screened with differential expression, COX and LASSO regression analyses. A prognostic signature was then established and assessed for its predictive value, stability and applicability using Kaplan-Meier analysis and receiver operating characteristic curves. The nomogram was further generated in combination with the signature and clinical characteristics. Characterization of immune properties and prediction of drug sensitivity were investigated on the signature-based subgroups using a panel of *in silico* platforms. Verification of gene expression was conducted with Western blot, qRT-PCR and ELISA, ultimately.

**Results:**

PPARG, LCK and LHX1 were identified and employed to construct the UbRGs-based prognostic signature, showing a strong ability to discriminate LC patients with distinct OS in TCGA-LC and GSE65858, and excellent applicability in most clinical conditions. The nomogram showed higher predictive value and net clinical benefit than traditional indicators. As evaluated, the low-risk group had a more activated immune function, higher infiltration of anti-cancer immune cells and stronger expression of immune-promoting cytokines than the high-risk group. Immune properties were also correlated with individual signature genes. PPARG and LHX1 were negatively correlated, whereas LCK positively correlated, with the immuno-promoting microenvironment. Additionally, chemotherapy would be more effective in high-risk patients, while immune checkpoint inhibitors would be more effective in low-risk patients. Finally, dysregulation of the signature genes was confirmed in LC cell lines by Western blot, and PPARG knockdown significantly reduced the expression of the immunosuppressive cytokines IL6, TGFB1, TGFB2 and VEGFC by qRT-PCR and ELISA.

**Conclusion:**

We have developed a UbRGs-based signature for LC prognostic evaluation that is valuable in clinical application, indicative of the immune microenvironment and beneficial for individualized treatment guidance.

## 1 Introduction

As the most common malignant tumor of the head and neck, there are approximately 188,960 new cases of laryngeal cancer (LC) and 103,216 related deaths annually worldwide, according to the latest GLOBOCAN report (Bray et al., 2024). With the application of comprehensive treatment strategies combining surgery, radiotherapy, chemotherapy and immunotherapy, the 5-year survival rate for certain LC patients has improved. However, the proportion of patients dying from recurrence, metastasis and resistance is still as high as 30%–40% (Steuer et al., 2017; Egelmeer et al., 2011). To reduce patient mortality, accurate prognostic prediction is essential for better survival estimation and optimization of therapeutic strategies. Current assessments depend primarily on the pathological characteristics of the tumor, especially the TNM stage. Unfortunately, the predictive power of the TNM stage is only 57% for overall survival (OS) and 60% for progression-free survival in LC patients (Cui et al., 2020a; Cui et al., 2020b). Obviously, current prognostic strategies have already hampered the accurate prediction of tumor progression and therapeutic response, and consequently will rarely support improvements in treatment. Therefore, to achieve better prognosis and efficacy in LC therapies, there’s an urgent need to establish new prognostic strategies and discover biomarkers of advantage.

It's well known that protein dysregulation and dysfunction are widespread in cancer cells (Díaz et al., 2021). As the pivotal regulatory machinery of protein homeostasis in eukaryotic cells, the ubiquitin-proteasome system is deeply involved in tumor initiation and progression (Sun et al., 2020). As reported by Wang et al., cell proliferation and radiotherapy resistance in LC were mediated by overexpression of UBR5, an E3 ubiquitin-protein ligase, through activation of the p38/MAPK signaling pathway (Wang et al., 2020). Another report on USP34, one of the deubiquitinating enzymes, indicated its role in enhancing LC cell growth and resistance to cisplatin by stabilizing SOX2 (Dai et al., 2020). In addition to direct effects on cancer cells, ubiquitin-related genes (UbRGs) also play an important role in facilitating cancer immune evasion (Çetin et al., 2021). For example, the E3 enzyme TRIM28 has been reported to induce the infiltration of myeloid-derived suppressor cells into small cell lung cancer, thereby promoting cancer progression through increased RIPK1 ubiquitination and activation of the downstream NF-κB pathway (Liang et al., 2023). During the anti-PD-1 treatments in colorectal cancer, its reactivity was impaired by a deubiquitinating enzyme USP14, which inhibited PD-1 expression and CD8^+^ T cell infiltration by targeting the IDO1/TRP/KYN signaling axis (Shi et al., 2022). In short, multiple properties of cancer will be altered by the disrupted expression of UbRGs through a panel of distinct mechanisms. As a consequence, patient survival and therapeutic response may be affected, suggesting that UbRGs could be employed as candidate biomarkers to develop novel strategies for predicting LC prognosis. However, the studies of UbRGs in LC are still insufficient, which hinders the understanding of their functional role and application in prognosis.

In this study, we aimed to develop a UbRGs-based prognostic signature and nomogram, attempting to achieve risk stratification and individualized survival prediction in LC patients. Multi-dimensional evaluations were then carried out to recognize the correlation between the UbRGs-based signature and the immune properties of the LC microenvironment. Subsequently, the potential regulatory role of the signature genes in LC immunity was thoroughly investigated by panels of *in silico* prediction and experiment validation. Finally, drug sensitivity prediction was performed to provide clues for the individualized therapy of LC patients based on this gene signature. Overall, our study was the first design of UbRGs-based prognostic signature of LC and provided new insights to improve prognosis prediction, understand cancer immunity, and guide individualized medication, which will ultimately shed new light on prolonging patient survival.

## 2 Materials and methods

**FIGURE 1:**
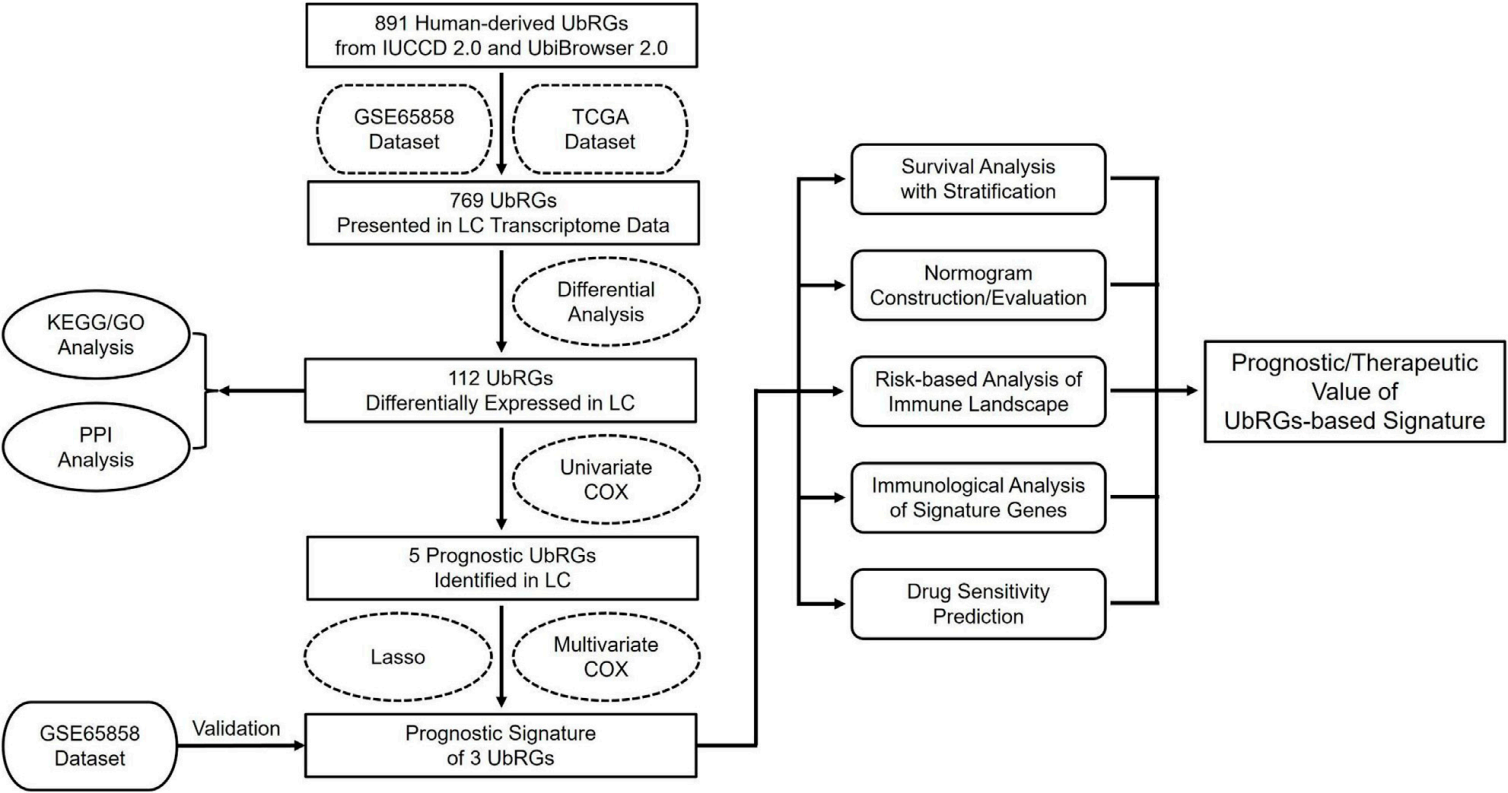
Flowchart of the current study.

The entire procedure of this study was summarized in the flowchart shown in Figure 1. All websites and calculation tools employed are listed in Supplementary Table S1.

### 2.1 Data collection and preprocessing

RNA-Seq data and clinical information of 116 LC and 12 normal laryngeal tissues were downloaded from The Cancer Genomic Atlas (TCGA). Expression profiling data were normalized using the transcripts per kilobase of exon model per million mapped reads format, and relevant clinical analyses were performed on 105 cases after excluding samples with missing pathological and survival information. The TCGA-LC dataset was defined as the training set. For signature validation, the GSE65858 dataset was downloaded from the Gene Expression Omnibus (GEO), from which the expression matrix and clinical data of 46 LC patients were extracted. Those two datasets were uploaded in Supplementary Data 1.

### 2.2 Identification and annotation of differentially expressed ubiquitin-related genes

Ubiquitin-related genes were collected from two databases, iUUCD 2.0 and UbiBrowser 2.0. Differentially expressed UbRGs (DUbRGs) were then screened from the harvested genes using the “limma” R package with criteria of FDR <0.05 and |log2 fold change (FC) | > 1. Both genes and samples were clustered using the “Complete” clustering method and the “correlation” distance calculation approach. Subsequently, a heatmap was generated to visually present the top 20 DUBRGs.

Functional analyses of DUbRGs were carried out with the Sangerbox 3.0 online platform for either the Kyoto Encyclopedia of Genes and Genomes (KEGG) or Gene Ontology (GO), including cellular components (CC), molecular functions (MF) and biological processes (BP). FDR <0.05 was considered as the significance threshold for the enrichment of candidate pathways. The analysis of potential protein-protein interaction (PPI) among DUbRGs was performed in STRING, with a minimum interaction score of 0.4. Visualization of the PPI network proceeded using Cytoscape software.

### 2.3 Construction and validation of a prognostic signature based on UbRGs

Univariate Cox regression analysis was used to preliminarily screen for DUbRGs that significantly correlated with overall survival (OS) based on the gene expression profile data of individuals in the training set. These DUbRGs were shrunk based on the minimum lambda determined by 10-fold cross-validation in the least absolute shrinkage and selection operator (LASSO) regression analysis. Genes with independent prognostic value were further identified with multivariate COX regression analysis among the ones resulting from univariate Cox regression. The expression value will then be termed as Exp and incorporated into the prognostic signature. Meanwhile, the coefficient of gene expression value was also generated in the same analysis and termed as β. This value was employed to quantify the contribution of each gene to the risk rate and thus more accurately reflect its weight in the overall assessment. Subsequently, the patient risk score was formulated as below:

$$\text{Risk score}=\sum_{i=1}^{n}\beta_{i}\times {\mathrm{Exp}}_{i}$$

Based on the median risk score, the individuals in the training set were divided into high- and low-risk groups. To assess the ability of the signature to discriminate OS in LC patients, Kaplan-Meier curves were plotted accordingly using the SRplot online platform. With this platform, receiver operating characteristic (ROC) curves were also plotted and the area under the curves (AUC) was calculated to evaluate the predictive efficacy of the signature in 1-, 2-, and 3-year OS of LC patients. In addition, the distribution characteristics were analyzed for the risk score, survival status and gene expression profiles. To assess the stability of the signature, the validation set was employed. The median risk score of the training set was also used as the basis to group high- and low-risk. The numbers of high- and low-risk patients in the training and validation sets are shown in Supplementary Table S2.

To evaluate the clinical applicability of the signature under different clinical characteristics, Kaplan-Meier analysis was conducted on the subgroups retrieved from the training set, including age (</> 60 years old), gender (male/female), differentiation grade (1-2/3-4), T stage (1-2/3-4), N stage (0-1/2-3), M stage (0/1) and clinical stage (I-II/III-IV). The number of high- and low-risk patients in each subgroup was shown in Supplementary Table S2.

### 2.4 Establishment and evaluation of nomogram

Univariate and multivariate COX regression analyses were performed on the combination of risk score and clinical characteristics in the training set. To convert the results of complex regression equations into simple graphs, a nomogram was constructed using the “regplot” R package, which can efficiently predict the probability of an individual’s outcome event according to the patient’s specific situation, thus achieving individualized assessment in clinics (Balachandran et al., 2015). ROC curves were generated to evaluate the predictive efficacy of the nomogram, risk score and traditional prognostic indicators (T stage, N stage, M stage and clinical stage) for 3-year OS in LC patients. Calibration curves were synthesized to assess the consistency between the predicted and actual survival rates using the “rms” R package. To evaluate the net clinical benefit of the nomogram and traditional stage, decision curve analysis (DCA) was performed using the “ggDCA” R package.

### 2.5 Gene set enrichment analysis (GSEA)

KEGG- and GO-related gene sets were downloaded from the GSEA. To explore potentially enriched biological functions in the high- and low-risk groups, GSEA enrichment analysis was carried out on the training set using the R packages termed “limma” and “clusterProfiler”. The enriched items were recognized with *p*-values lower than 0.05 and then ranked based on NES resulting from the normalization of enrichment scores. The top 5 enriched items were visualized as curves in GSEA plots, while pie charts were used to show the categories and percentages within all enriched items.

### 2.6 Analysis of immune landscape

To understand the potential correlation between the UbRGs-based prognostic signature and cancer immunity in LC, a panel of immune properties was calculated for individuals in the training set and then compared between the high- and low-risk groups as follows.

1) The activation degree of 13 immune-related pathways was assessed with the ssGSEA algorithm.
2) The TME scores (including stromal score, immune score and ESTIMATE score) were calculated using the ESTIMATE algorithm to determine the proportion of TME cells in LC.
3) The infiltration levels of immune cells were estimated using the CIBERSORT and ssGSEA algorithms, and the correlation between risk score and infiltration level of each cell type was further analyzed with the Chiplot online platform.
4) The expression levels of immune-related cytokines were analyzed using the Sangerbox 3.0 online platform.

### 2.7 Cell culture and CRISPR-based gene knockout

Human LC cell lines (TU686, TU212 and LCC) and a normal lung epithelial cell line (Bease-2B) were purchased from Meilun, Yihe, QuiCell and Aorisai Biotechnology Co., LTD respectively. They were cultured in RPMI 1640 or DMEM-H medium containing 10% fetal bovine serum and 1% penicillin/streptomycin. Cells were passaged at a ratio of 1:3 ratio upon reaching 80% confluence.

Plasmids containing control or PPARG-targeting sgRNAs were constructed based on the LentiCRISPR v2 vector (Addgene #52961) for stable gene knockout in cell lines. The lentivirus was then packaged into 293T cells and applied to infect TU212 and TU686 cells. After infection, continuous puromycin selection was performed at a concentration of 2 μg/mL to obtain stable cell lines for further experiments. The sgRNA sequences are listed in Supplementary Table S3.

### 2.8 Western blot (WB)

Western blot assays were performed using routine methods. Briefly, the cells were lysed with RIPA buffer on ice once reaching 80% confluence. After centrifugation at 4°C, 12,000 rpm for 30 min, protein samples were collected from the supernatant, mixed with 1×loading buffer, and then heated at 95°C for 30 min. The heated samples were further subjected to SDS-PAGE electrophoresis and antibody staining.

The expression levels of PPARG, LCK and LHX1 proteins were determined in TU686, TU212, LCC and Bease-2B cells. The knockout efficiency of PPARG was tested in TU212- and TU686-derived control and PPARG knockout cells. Hence, the primary antibodies used in this study included PPARG (Proteintect, 66936-1-Ig), LCK (Abcam, ab227975), LHX1 (Santa, sc-515631) and GAPDH (Proteintect, 66936-1-IG). 10494-1-AP). Goat Anti-Rabbit IgG-HRP (Affinity, S0001) and Goat Anti-Mouse IgG-HRP (Affinity, S0002) were adopted as secondary antibodies. The grayscale values of the protein bands were analyzed semi-quantitatively using ImageJ software.

### 2.9 Quantitative real-time polymerase chain reaction (qRT-PCR)

qRT-PCR was performed to analyze the mRNA expression levels of cytokines, including IL1A, IL6, IL18, CXCL11, CCL2, VEGFC, TGFB1, TGFB2, TGFB3, CSF1, FGF2 and PDGFC, in control and PPARG knockout groups derived from TU212 and TU686 cell lines.

Total RNA extraction, cDNA synthesis and qRT-PCR reaction were performed according to the kit manuals (Vazyme, R701-01; Takara, RR036A and RR820A). GAPDH was hired as the internal reference gene. Relative mRNA levels of cytokines were calculated by the 2^−ΔΔCT^ method. Primer sequences are listed in Supplementary Table S4.

### 2.10 Enzyme-linked immunosorbent assay (ELISA)

Following the qRT-PCR results, the protein expression levels of IL6, VEGFC, TGFB1 and TGFB2 in the supernatants of control and PPARG knockout cells were examined using ELISA. The ELISA kits (RUIXIN BIOTECH; RX106126H, RX105005H, RX104768H, RX2D118026) were equilibrated at room temperature before the experiment. Operations were then performed following the instructions and the OD values were detected at 450 nm. The standard curves were subsequently plotted and the protein concentrations were calculated from the corresponding OD values.

### 2.11 Prediction of drug sensitivity in LC patients

The response of LC patients to immunotherapy was forecasted using the TIDE algorithm, and the expression levels of 48 immune checkpoints were further assessed with the Sangerbox 3.0 online platform.

Half inhibitory concentrations (IC50) for chemotherapy and targeted drugs were predicted using the “oncoPredict” R package. Briefly, drug sensitivity data valued as IC50 of tumor cell lines was retrieved from the Genomics of Drug Sensitivity in Cancer (GDSC) database, whereas the corresponding gene expression profiles were also obtained from the same source. They were used to build ridge regression models, which were applied on the training set mentioned in Section 2.1 to yield drug sensitivity predictions. These drug models were built after removing or summarizing gene duplication, homogenization (batch correction), and filtering low-variant genes. Subsequently, calcPhenotype function was applied to the proceeded, standardized and filtered clinical tumor expression data, yielding a drug sensitivity prediction for each patient. Averaged IC50 was ultimately calculated in either high- or low-risk groups respectively for each drug.

### 2.12 Statistical analysis

All statistical analyses were performed using R software (version 4.3.2), GraphPad Prism (version 9.0) and the online platforms mentioned above. Differences in gene expression, immune infiltration and IC50 predictions across the database were compared using the Wilcoxon signed-rank test. Spearman was employed for the correlation analysis. One-way ANOVA was used to compare differences in protein expression of signature genes among cell lines. The significance of differential cytokine expression was confirmed by t-test. *p* < 0.05 was considered statistically significant.

## 3 Results

### 3.1 A prognostic signature was constructed based on PPARG, LCK and LHX1 highlighted from UbRGs differentially expressed in LC

**FIGURE 2:**
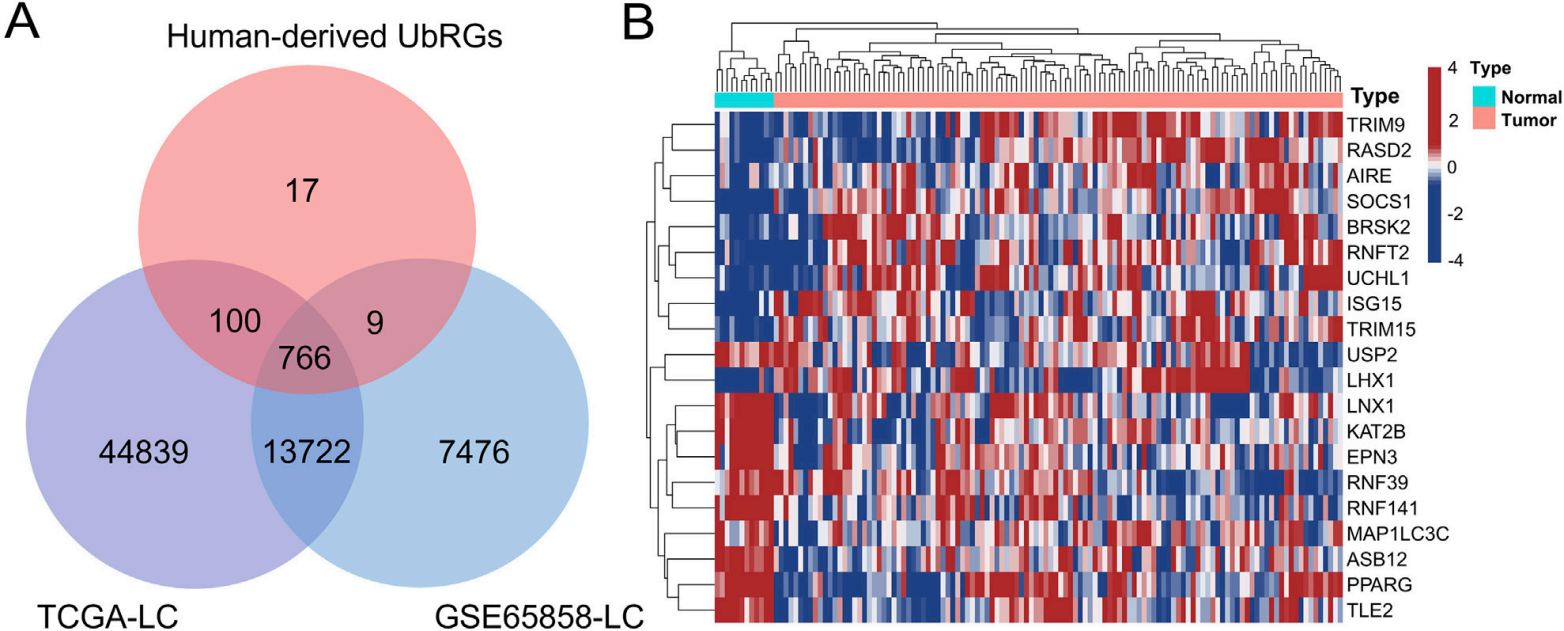
Screening of UbRGs differentially expressed in LC. **(A)** The Venn diagram showed 769 UbRGs retrieved from human-derived databases and presented in the TCGA-LC and GSE65858-LC datasets. **(B)** The heatmap of the top 20 differentially expressed UbRGs, with both genes and patients clustered.

Initially, 1366 UbRGs were retrieved from iUUCD 2.0, including 27 E1 enzymes, 109 E2 enzymes, 1153 E3 enzymes, 164 deubiquitinating enzymes (DUBs), 396 ubiquitin/ubiquitin-like binding domains and 183 ubiquitin-like domains. Meanwhile, 417 E3 enzymes and 86 DUBs were obtained from UbiBrowser 2.0. After removing duplicates and non-human records, 891 human-derived UbRGs were obtained in combination (Supplementary Date 2). Of these, expression data were extracted for 766 UbRGs from both the training and validation sets (Figure 2A). 111 UbRGs were shown as differentially expressed between LC and normal laryngeal tissues in the training set, containing 100 upregulated and 11 downregulated ones, which were termed DUbRGs. The top 20 DUbRGs were presented in the heatmap (Figure 2B). The potential biological functions and protein interactions of these DUbRGs were revealed by KEGG, GO and PPI analyses (Supplementary Figure S1).

**FIGURE 3:**
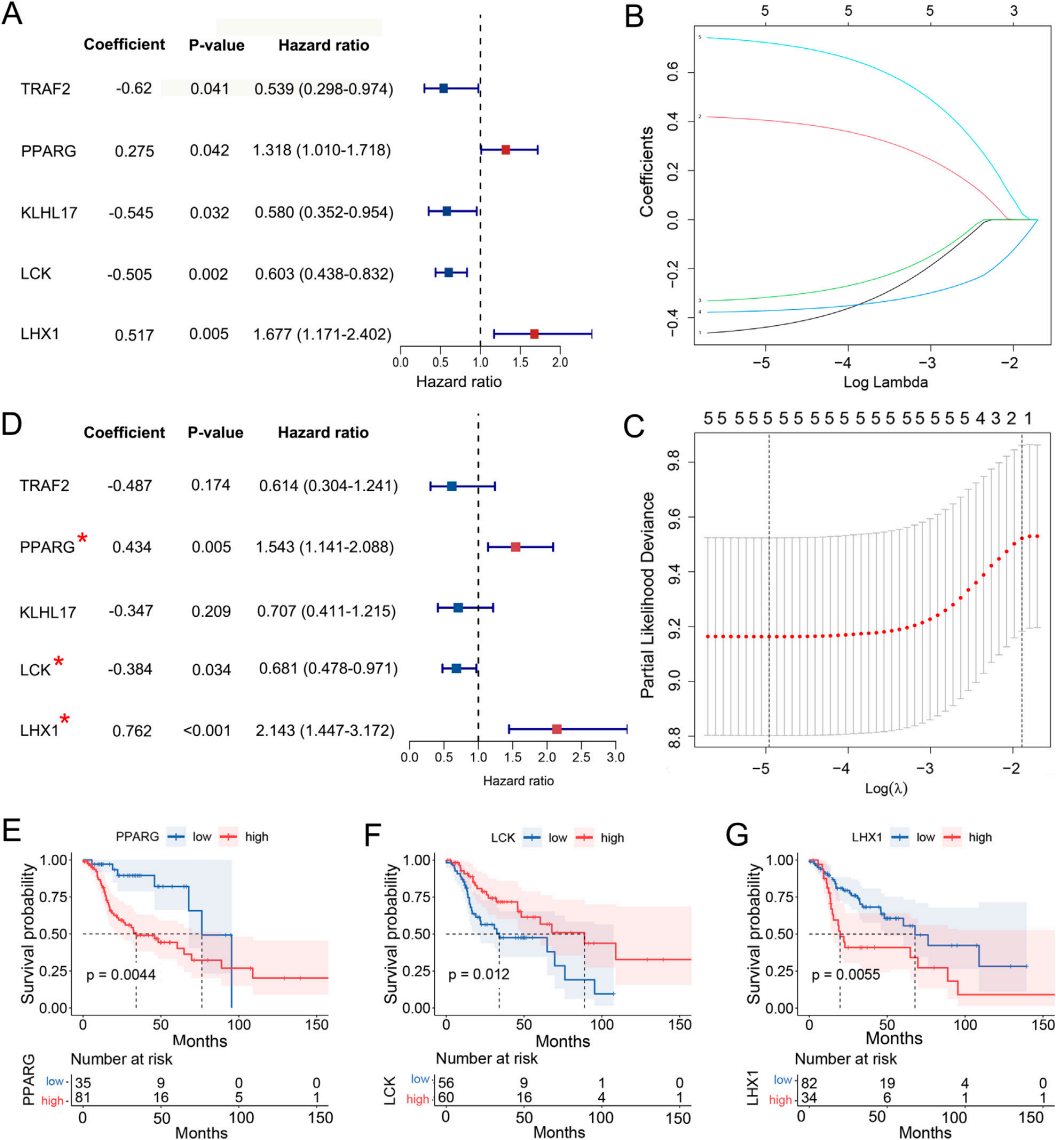
Identification of 3 UbRGs to construct the prognostic signature. **(A)** Univariate COX regression analysis of 5 UbRGs significantly associated with OS in LC patients, including TRAF2, PPARG, KLHL17, LCK and LHX1. **(B)** Coefficient profiles of these five UbRGs generated with LASSO regression analysis. **(C)** Determination of minimum lambda for 5 via 10-fold cross-validation in LASSO regression. **(D)** Multivariate Cox regression analysis of 3 UbRGs in-dependently affected OS in LC patients, including PPARG, LCK and LHX1. **(E–F)** Kaplan-Meier analysis of identified UbRGs, **(E)** PPARG, **(F)** LCK and **(G)** LHX1.

Out of 111 DUbRGs, 5 genes were highlighted with significant correlation to the OS of LC patients in univariate COX regression, including TRAF2, PPARG, KLHL17, LCK and LHX1 (Figure 3A). To avoid model overfitting, LASSO regression analysis was performed and these 5 DUbRGs remained when applying the minimum lambda (Figures 3B, C). To further determine the DUbRGs that independently influenced OS, multivariate Cox regression analysis was conducted. PPARG, LCK and LHX1 were subsequently identified as the genes highly correlated with the prognosis of LC patients (Figure 3D), while the other two genes were excluded due to *p* > 0.05. Of these, PPARG and LHX1 were indicated as risk genes with coefficients valued at 0.434 and 0.762 respectively, whereas LCK was indicated as a protective gene with a coefficient of −0.384. Also, such identity was confirmed by Kaplan-Meier analysis (Figures 3E–G). The shorter survival was correlated with high expression of PPARG and LHX1, while with low expression of LCK. Their differential expression in LC and normal lung epithelial cell lines was verified by Western blot, which was consistent with the RNA-seq results (Supplementary Figure S2).

A UbRGs-based prognostic signature was thus established as described in Section 2.3, where the risk score was formulated based on both gene expression levels and their corresponding coefficients:

Risk Score=0.434×PPARG+0.762×LHX1 ‐ 0.384×LCK

### 3.2 The effectiveness of UbRGs-based signature was proved and a related nomogram was established accordingly

**FIGURE 4:**
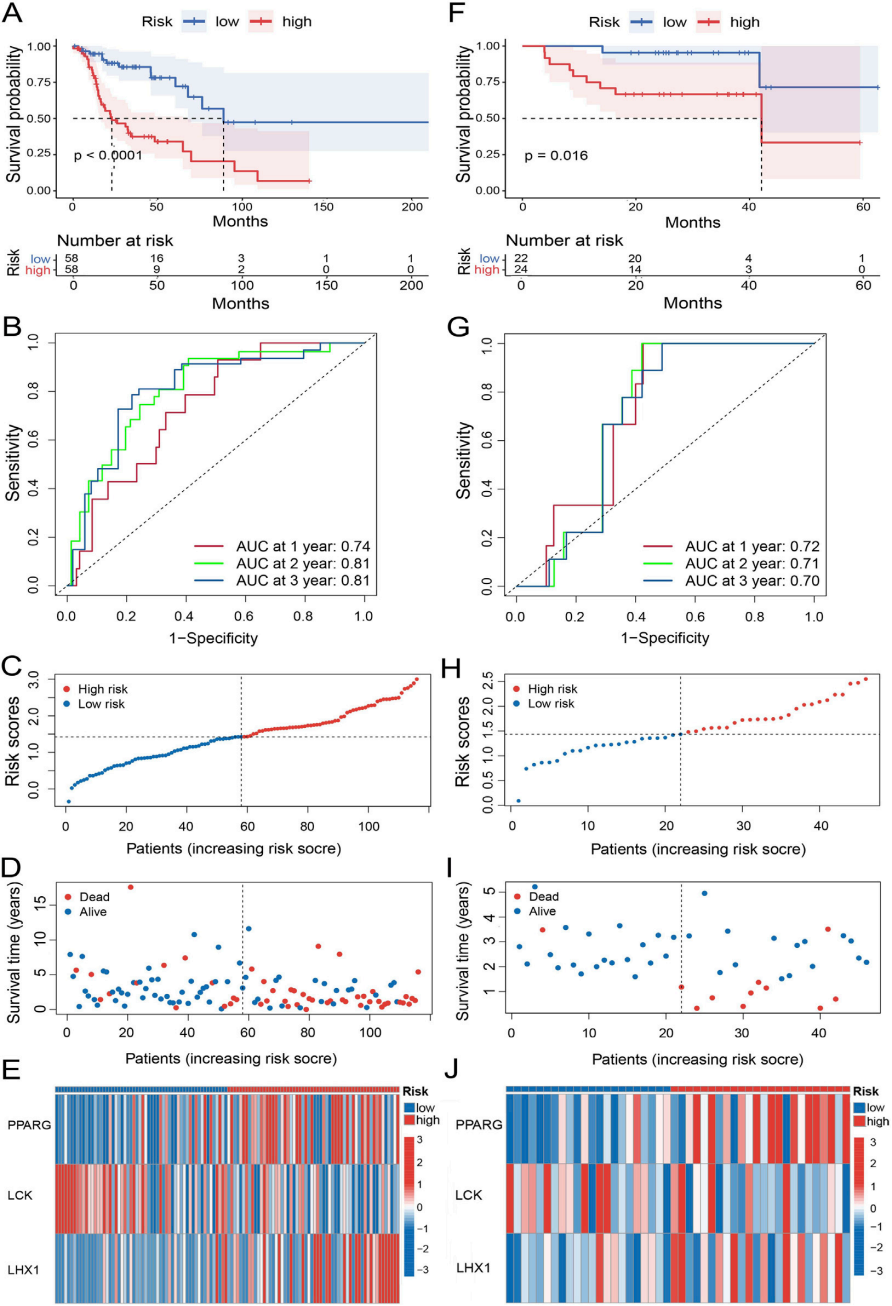
Evaluation of the prognostic performance of the UbRGs-based signature. **(A)** Kaplan-Meier analysis of OS in the high- and low-risk groups based on the training set. **(B)** ROC curves showing the predictive efficacy of this signature for 1-, 2-, and 3-year OS of patients in the training set. **(C–E)** Distribution of characteristics of individual patients in the training set, in-cluding **(C)** risk score, **(D)** survival status, and **(E)** expression profiles of 3 signature genes. **(F–J)** Evaluation of the UbRGs-based signature with the validation set for **(F)** Kaplan-Meier analysis of OS, **(G)** ROC curves of predictive efficacy in 1-, 2-, 3-year OS, and **(H)** risk score, **(I)** survival status, and **(J)** expression profiles of 3 signature genes in individual patients.

To evaluate the effectivity of the UbRGs-based prognostic signature, a panel of calculations was carried out on the training set. At first, the median risk score was determined as 1.43 by the formulation in **3.1**, with which the high- and low-risk groups were then divided from the training set. The median survival time was defined as 22.85 months for the high-risk group and 88.87 months for the low-risk group with Kaplan-Meier analysis (*p* < 0.0001, Figure 4A), which demonstrated a significant difference in OS between the two groups. With the ROC curves of 1-, 2-, and 3-year OS, AUC values were calculated as 0.74, 0.81, and 0.81 respectively, indicating a good predictive efficacy of this signature (Figure 4B). Along with the increasing risk score (Figure 4C), the analysis of individual patients showed higher mortality (Figure 4D). Meanwhile, the expression levels of the risk genes, PPARG and LHX1, tended to be upregulated in high-risk patients; while the trend of protective gene LCK was downregulated (Figure 4E).

To assess the stability of the UbRGs-based signature, the same panel of analyses was subsequently conducted in the validation set. Worse OS was still significantly observed in the high-risk group (p = 0.016, Figure 4F). The AUC values were 0.72, 0.71 and 0.70 for 1-, 2-, and 3-year OS in LC patients respectively (Figure 4G), further confirming the effectiveness of the prediction. The correlation between the risk score and mortality as well as the expression trend of the signature genes were in good agreement with the results from the training set (Figures 4H–J).

**FIGURE 5:**
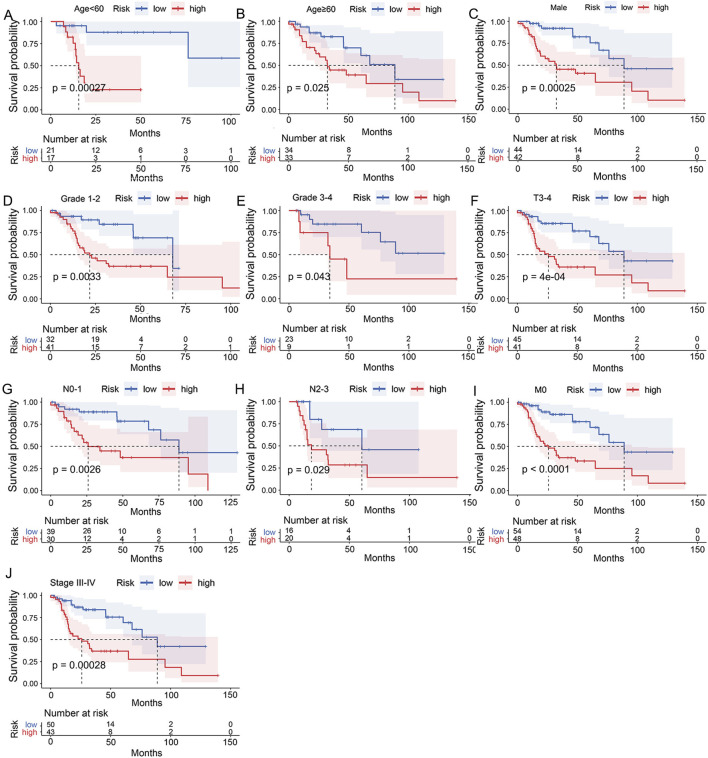
Assessment of the clinical applicability of the UbRGs-based signature. Significant dif-ferences in OS were exhibited between the high- and low-risk groups in conditions of **(A)** age<60, **(B)** age≥60, **(C)** male, **(D)** Grade 1-2, **(E)** Grade 3-4, **(F)** T3-4 stage, **(G)** N0-1 stage, **(H)** N2-3 stage, **(I)** M0 stage and **(J)** clinical stage III-IV by stratified Kaplan-Meier analysis.

To investigate the applicability of the UbRGs-based signature, Kaplan-Meier analysis was carried out across a range of clinical conditions. In the majority of cases (Figures 5A–J), shorter OS was significantly correlated with the high-risk group (*p* < 0.05), including age<60, age≥60, male, Grade 1-2, Grade 3–4, T3-4 stage, N0-1 stage, N2-3 stage, M0 stage and clinical stage III-IV. However, in the other four conditions (Supplementary Figure S3), namely, female (*p* = 0.087), T1-2 stage (*p* = 0.083), M1 stage (*p* = 0.16) and clinical stage I-II (*p* = 0.13), there was no significant difference in survival between the two groups.

**FIGURE 6:**
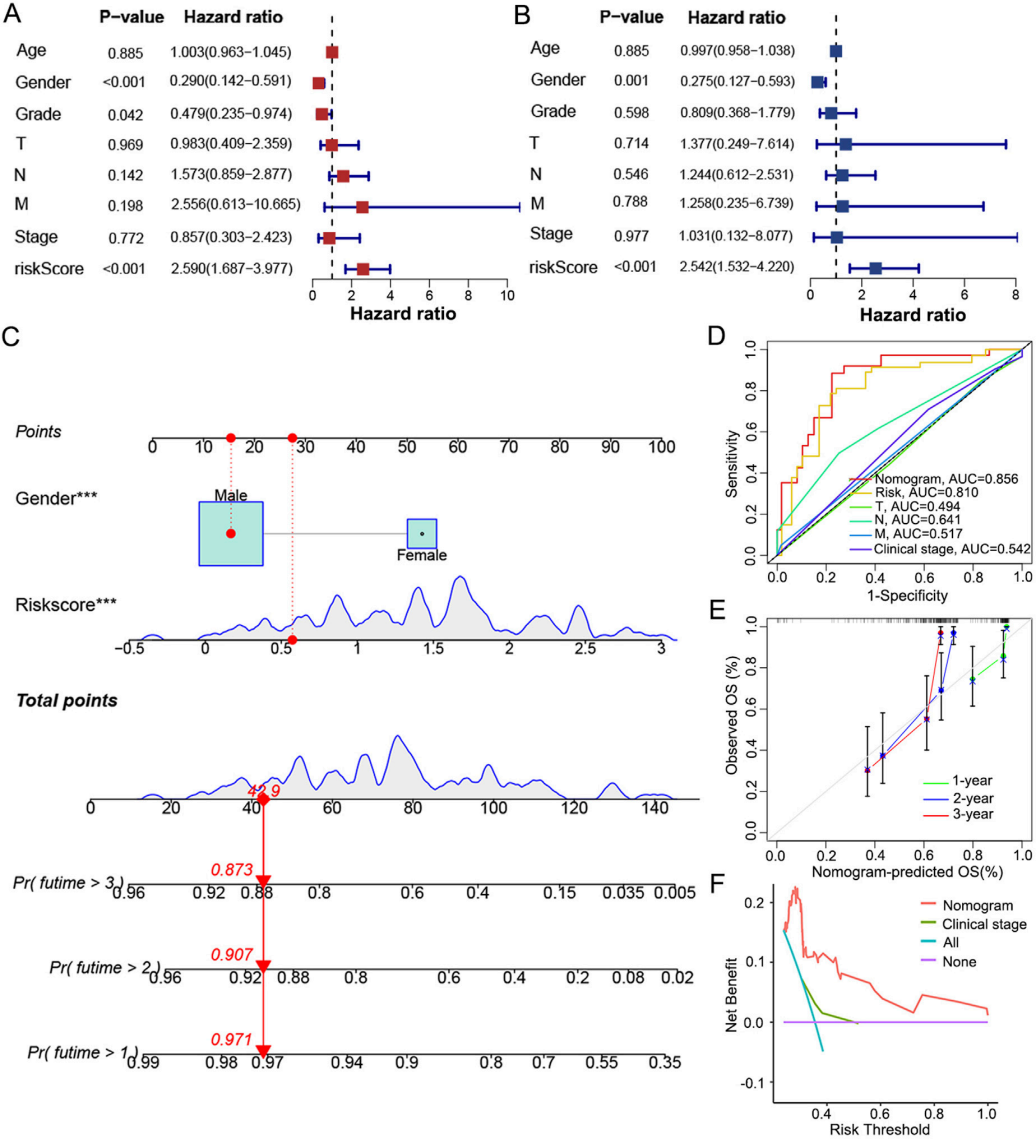
Establishment and evaluation of a nomogram integrating signature with gender. **(A)** Univariate and **(B)** multivariate COX regression analysis to highlight independent factors af-fecting OS in LC patients. **(C)** A nomogram constructed to predict the 1-, 2-, and 3-year OS in LC patients by combining the risk score with gender. **(D)** ROC curves based on the nomogram, risk score and traditional indicators to show the predictive efficacy of 3-year OS in LC patients. **(E)** Calibration curves of the consistency between the predicted and actual survival rates at 1, 2 and 3 years. **(F)** DCA showed the net clinical benefit of the nomogram and traditional stage.

To achieve individualized prediction, univariate and multivariate COX regression analyses were performed on the risk score and clinical characteristics. As the risk score (*p* < 0.001) and gender (*p* = 0.001) showed independent values in prognosis (Figures 6A, B), a nomogram was then constructed accordingly to visualize these results (Figure 6C). The probability of survival at 1, 2 and 3 years can be predicted more intuitively based on an individual’s risk score and gender profile. When comparing the ROC curve of 3-year OS in LC patients, the AUC of the nomogram was 0.856, higher than that of the risk score (AUC = 0.810) and traditional indicators (T stage, AUC = 0.494; N stage, AUC = 0.641; M stage, AUC = 0.517; clinical stage, AUC = 0.542; Figure 6D). With the calibration curve, good consistency was indicated between the predicted and actual survival rates of the nomogram at 1, 2, and 3 years (Figure 6E). Additionally, the DCA curve demonstrated the nomogram as a better predictive tool than the clinical stage (Figure 6F).

In brief, the signature composed of PPARG, LHX1 and LCK was shown efficacious in the prognosis prediction of OS in LC patients and applicable in most clinical conditions. The nomogram combining risk score and gender provided an even better predictive efficiency than the signature and traditional indicators alone.

### 3.3 The UbRGs-based signature specified the status of the immune microenvironment in LC

**FIGURE 7:**
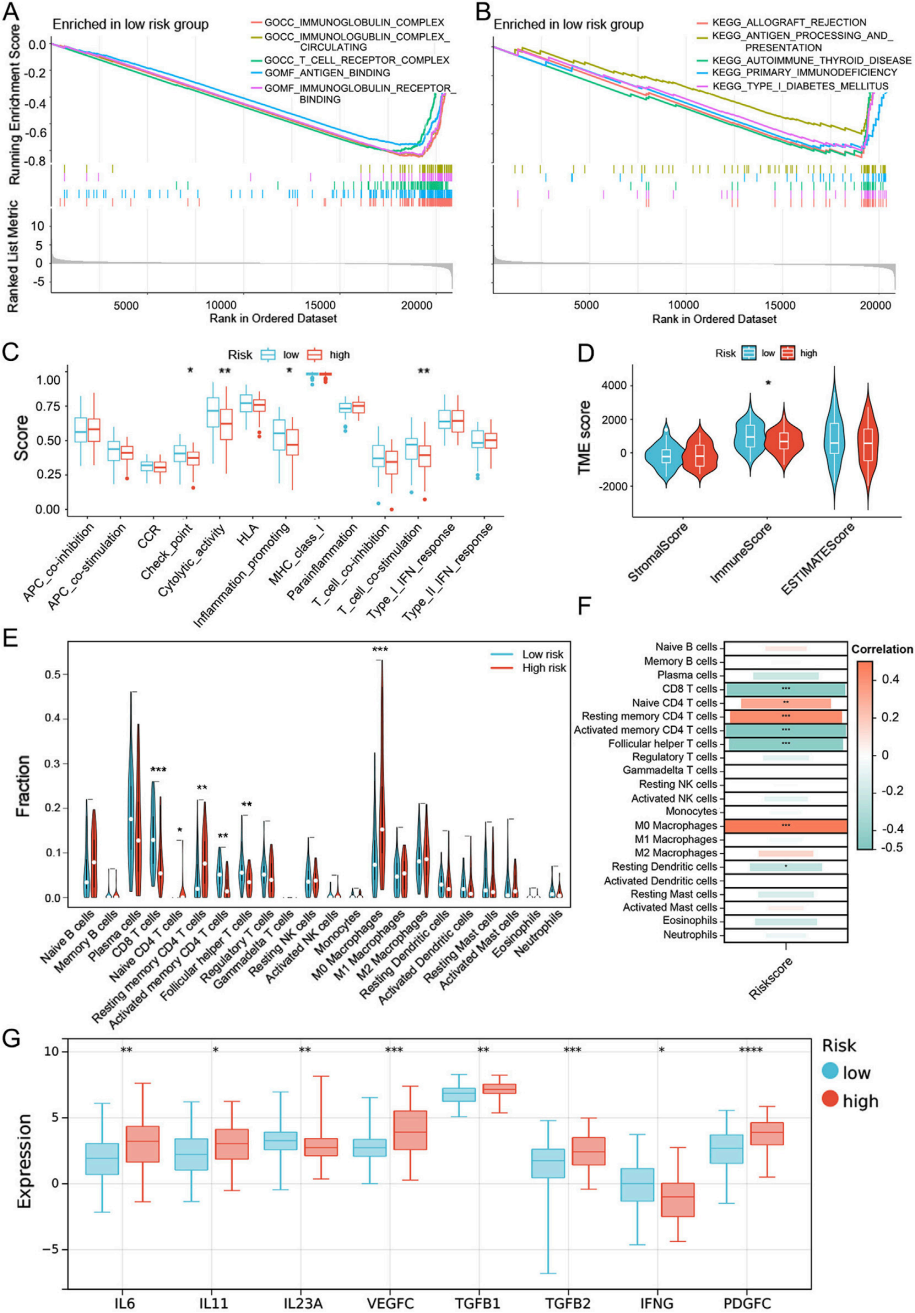
Evaluation of the immune microenvironment in the high- and low-risk groups dis-criminated by the UbRGs-based signature. **(A, B)** GSEA enrichment analysis in the low-risk group based on **(A)** GO- and **(B)** KEGG-related gene sets. **(C)** Differential activation of immune-related pathways in the high- and low-risk groups analyzed with the ssGSEA algorithm. **(D)** TME scores calculated by the ESTIMATE algorithm for both groups, in which the immune score was signifi-cantly different. **(E)** Estimation of immune cell infiltration levels via the CIBERSORT algorithm and 6 cell types highlighted with statistical significance. **(F)** Correlation between risk score and immune cell infiltration. **(G)** Immune-related cytokines differentially expressed between the high- and low-risk groups as predicted by the Sangerbox 3.0 online platform. ^*^ *p* < 0.05; ^**^ *p* < 0.01; ^***^ *p* < 0.001; ^****^ *p* < 0.0001.

For a better understanding of the UbRGs-based signature in prognosis prediction, biological processes differentially involved in the high- and low-risk groups were searched with GSEA enrichment analysis. By *p* < 0.05, 934 items were enriched with GO and 31 with KEGG (Supplementary Data 3). A prominent panel of immune-related processes was highlighted in the low-risk group. The top 5 enriched GO items included antigen processing and presentation, T-cell receptor complex, antigen binding, immunoglobulin receptor binding and immunoglobulin complex circulating (Figure 7A). Furthermore, 18 among top 20 enrichment in GO, and also 26 among top 30, were occupied by the immune-related processes (Labeled in Supplementary Data 3 with yellow). A similar trend of enrichment was also highlighted in KEGG items, with top 5 enriched biological processes were: allograft rejection, type I diabetes mellitus, autoimmune thyroid disease, primary immunodeficiency, and antigen processing and presentation (Figure 7B, Supplementary Data 3). When the immune-related items were counted in all GO and KEGG enrichments, as visualized in Supplementary Figures S4A–D, high percentages were quantified as 46.15% in GO-CC, almost 100% in GO-MF, 74.01% in GO-BP and 69.23% in KEGG. However, in the high-risk group, it was failed to summarize a dominant module with clear and unique functional connotation from the GSEA results, either by GO or KEGG (Supplementary Figures S4E–H), especially, no significant enrichment of immune processes observed (Supplementary Figures S5A, B).

To investigate whether the effectiveness of the UbRGs-based signature was due to the distinct immune status in LC, the immune landscape was further explored in multiple dimensions. At first, functional groups of immune-related pathways were analyzed with the ssGSEA algorithm. In the low-risk group, functional modules of checkpoint, cytolytic activity, pro-inflammatory and T-cell co-stimulation were preferentially activated, whereas no functional modules were shown dominant in the high-risk group (Figure 7C). TME scores were then calculated for individuals with the ESTIMATE algorithm. Of the three TME scores, the immune score is the only one significantly higher in the low-risk group, rather than the stromal score and ESTIMATE score (Figure 7D), which confirmed a higher degree of immune cell infiltration in low-risk LC patients. With the CIBERSORT and ssGSEA algorithms, the infiltration level of each immune cell type was specifically speculated. The cell types with anti-tumor effects showed a higher degree of infiltration in the low-risk group (e.g., CD8 T cells, activated memory CD4 T cells, follicular helper T cells, activated B cells and natural killer T cells), while primitive or resting immune cell types (e.g., native CD4 T cells, resting memory CD4 T cells and M0 macrophages) were dominant in the high-risk group (Figure 7E; Supplementary Figure S5C). The correlation between cell types and risk score was consistent with the trend of immune cell infiltration (Figure 7F). As immune regulation was generally mediated by cytokines, the expression preference of cytokines was also analyzed. Higher levels of immune-promoting cytokines IL23A and IFNG were predicted in the low-risk group, whereas immune-suppressing cytokines (e.g., IL6, IL11, VEGFC, TGFB1, TGFB2 and PDGFC) were preferentially expressed in the high-risk group (Figure 7G).

With a series of analyses, significant differences in the immune landscape were exhibited between the high- and low-risk groups, which was potentially one of the major origins of distinct outcomes in clinics and suggested the signature genes as immune regulators in LC.

### 3.4 Signature genes PPARG, LHX1 and LCK involved in sculpturing the LC immune microenvironment

**FIGURE 8:**
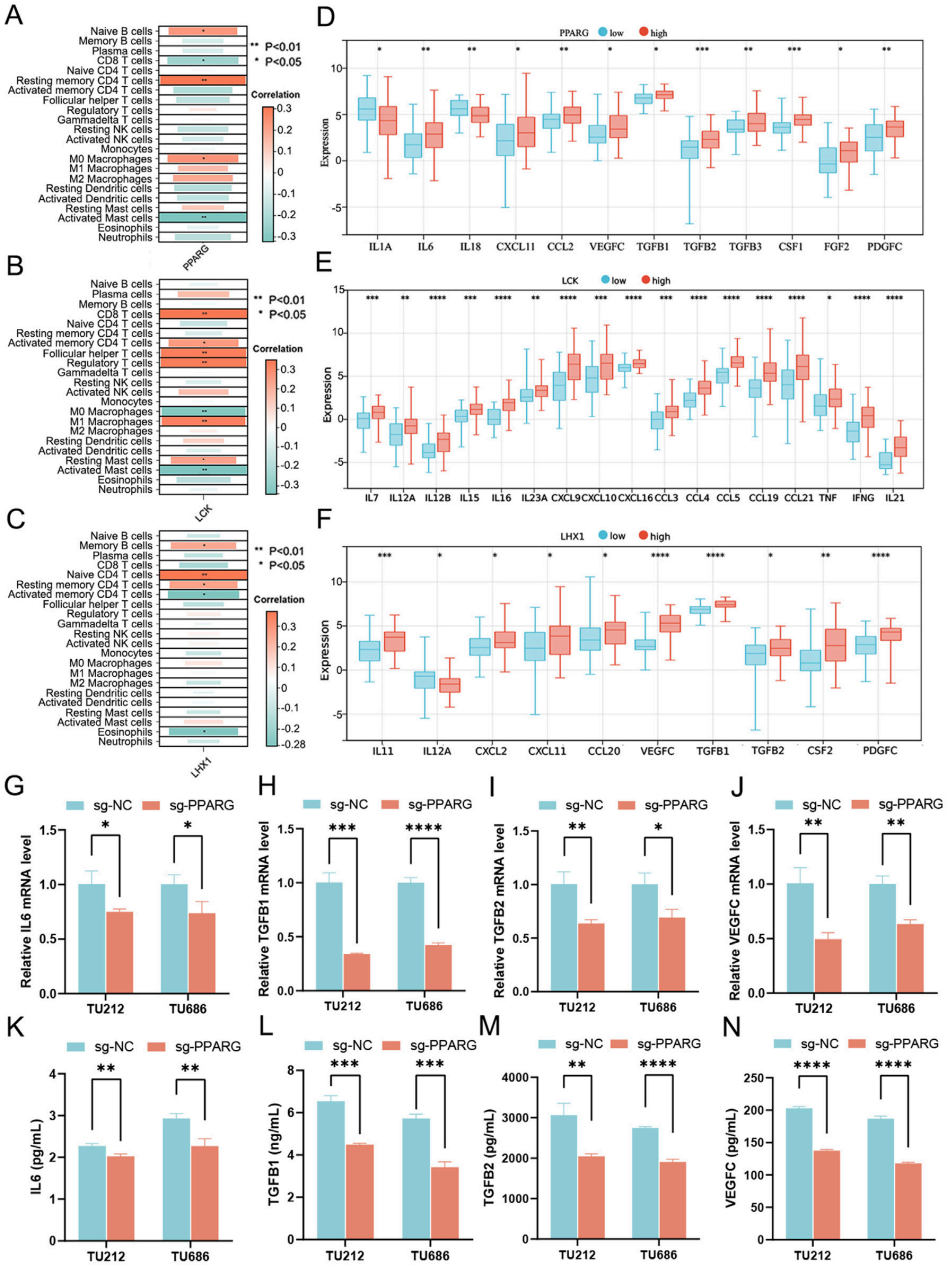
Investigation of the role played by signature genes in sculpting the immune landscape. **(A–C)** Correlation between immune cell infiltration and expression levels of 3 signature genes, **(A)** PPARG, **(B)** LCK and **(C)** LHX1. **(D–F)** Relationship between expression levels of immune-related cytokines and 3 signature genes, **(D)** PPARG, **(E)** LCK and **(F)** LHX1. **(G–I)** Cytokine mRNA levels quantified by qRT-PCR in PPARG knockout cells derived from LC cell lines TU212 and TU686, including **(G)** IL6, **(H)** TGFB1, **(I)** TGFB2 and **(J)** VEGFC. **(K–N)** Cytokine secretion levels meas-ured using ELISA in the supernatant of PPARG knockout LC cells, including **(K)** IL6, **(L)** TGFB1, **(M)** TGFB2 and **(N)** VEGFC. ^*^ *p* < 0.05; ^**^ *p* < 0.01; ^***^ *p* < 0.001; ^****^ *p* < 0.0001.

To confirm the regulatory role of signature genes in the immunity of LC, the degree of immune cell infiltration and expression levels of cytokines relevant to each gene were predicted one by one. It was clearly visualized that the risk genes PPARG and LHX1 were negatively correlated with anti-tumor effectors (e.g., CD8 T cells and activated memory CD4 T cells), positively correlated with primitive or resting immune cells (e.g., naive B cells, memory B cells, naive CD4 T cells, resting memory CD4 T cells and M0 macrophages), while the protective gene LCK was positively correlated with anti-tumor effectors (CD8 T cells, activated memory CD4 T cells, follicular helper T cells and M1 macrophages; Figures 8A–C). Among the cytokines, immune activators (e.g., IL1A, IL18 and IL12A) were at lower levels and immune suppressors (e.g., IL6, CXCL11, CCL2, VEGFC, TGFB1, TGFB2, TGFB3, CSF1, FGF2, PDGFC, IL11, and CCL20) were at higher levels when PPARG and LHX1 were highly expressed (Figures 8D, F). Besides, another group of immune activators (e.g., IL7, IL12, IL15, IL16, IL23A, CXCL9, CXCL10, CXCL16, CCL3, CCL4, CCL5, CCL19, CCL21, TNF, IFNG, IL21) were overexpressed along with higher expression of LCK (Figure 8E).

Since PPARG showed the highest node index among three signature genes in the PPI network of DUbRGs (Supplementary Figure S1C), further validation was then carried out with PPARG knockout cells generated from TU212 and TU686 cell lines (Supplementary Figures S6A–D). 12 immune-related cytokines were assessed with qRT-PCR, including IL1A, IL6, IL18, CXCL11, CCL2, VEGFC, TGFB1, TGFB2, TGFB3, CSF1, FGF2 and PDGFC, which were predicted in relevance with PPARG expression. At the mRNA level, four immunosuppressive cytokines (IL6, TGFB1, TGFB2 and VEGFC) showed significant downregulation in both PPARG knockout cell lines (Figures 8G–J). Simultaneously, their secretion levels in the supernatant were decreased (Figures 8K–N), as confirmed using ELISA. The other four (e.g., CCL2, CSF1, PDGFC and TGFB3) were downregulated in PPARG knockout TU212 cells (Supplementary Figures S6E–H), while the rest (e.g., FGF2, IL1A, IL18 and CXCL11) mostly showed no significant change (Supplementary Figures S6I–L), as quantified by qRT-PCR.

### 3.5 The UbRGs-based signature provided insights to the personalized therapies in clinics

**FIGURE 9:**
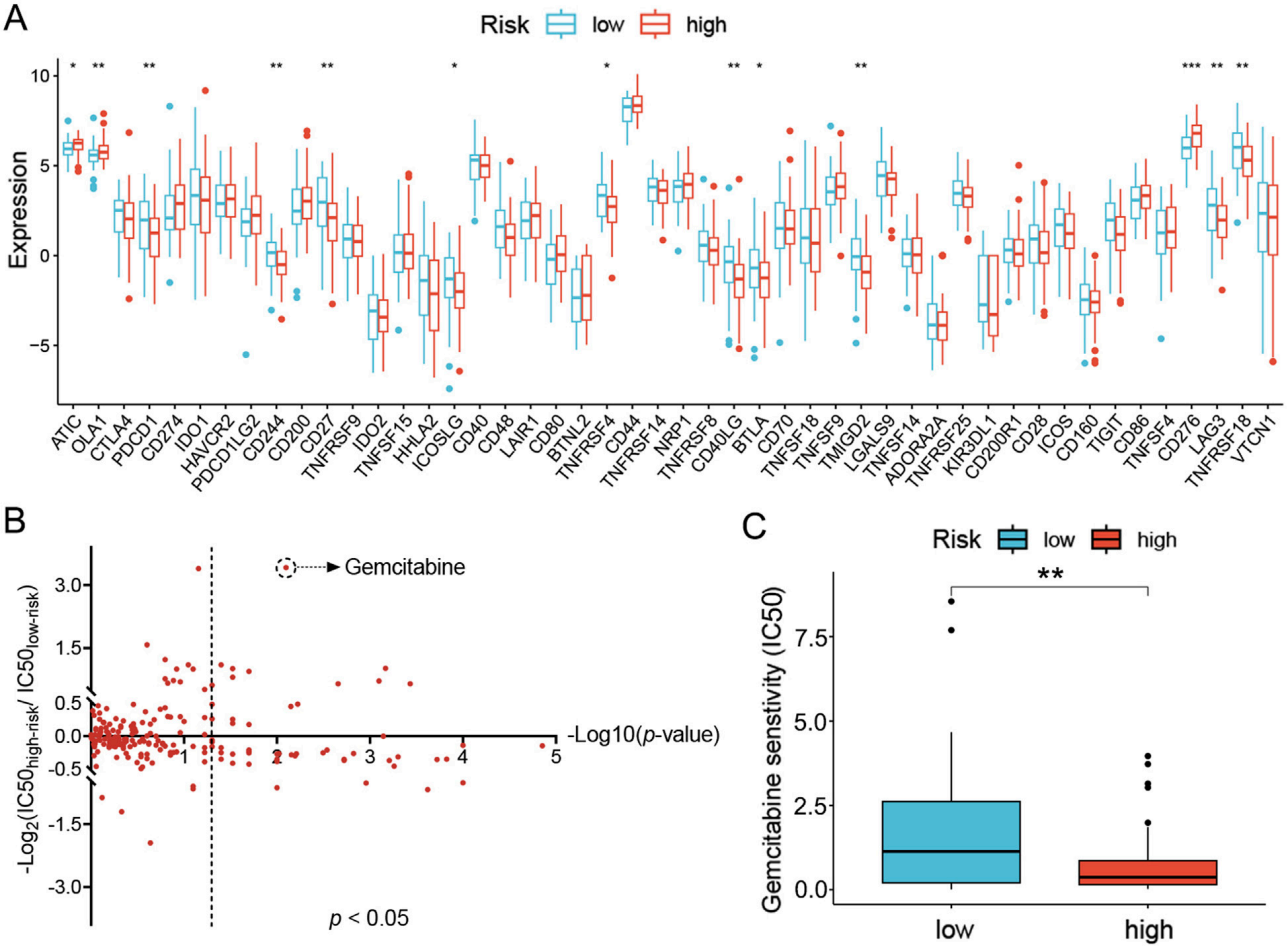
Prediction of sensitivity to clinical treatments for the high- and low-risk groups of LC patients. **(A)** Immune checkpoints differentially expressed in the high- and low-risk groups. **(B)** Predicted sensitivity to chemotherapy and targeted agents in the high- and low-risk groups. Data were plotted as -Log10(*p*-value) on the x-axis and -Log2(IC50_high-risk_/IC50_low-risk_) on the y-axis. **(C)** Predicted IC50 for gemcitabine in both groups. ^*^ *p* < 0.05; ^**^ *p* < 0.01; ^***^ *p* < 0.001.

Since the distinct immune landscape in LC had been defined with the UbRGs-based signature and applied to the prognosis of OS, its instructiveness for immunotherapy was subsequently investigated. With the TIDE algorithm, a slight trend of higher response to immunotherapy was predicted in the low-risk group (41% vs. 31%), but unfortunately, no significance in statistics was shown (Supplementary Figure S7A). However, differential expression of immune checkpoint genes was exhibited with the ssGSEA algorithm (Figure 7C). The vast majority of immune checkpoints (e.g., PDCD1, CD244, CD27, ICOSLG, TNFRSF4, CD40LG, BTLA, TMIGD2, LAG3 and TNFRSF18) presented significant elevation of expression in the low-risk group, while only a few (e.g., ATIC, OLA1 and CD276) in the high-risk group (*p* < 0.05, Figure 9A). The differential expression data suggested that immune checkpoint inhibitor treatment may be more effective for LC patients with lower risk scores.

Alternatively, sensitivity to chemotherapy and targeted therapeutic agents was also analyzed. The reference tumor cell lines recorded in GDSC were employed as a reference to correlate gene expression signature and IC50 of drugs in a quantitative model. In high- and low-risk groups, the sensitivity to each drug was then evaluated via comparing the gene expression profiles of patients in either group with the reference tumor cell lines. The resultant IC50 values were presented in a 2D format similar to the volcano plot. For each drug, the Y-coordinate presented the -Log2(IC50_high-risk_/IC50_low-risk_) value to show the difference of IC50 values between the two groups, while the -Log10(p-value) was projected as the X-coordinate to indicate the significance of the difference. A total of 48 agents were identified with a significant difference in sensitivity between the high- and low-risk groups (*p* < 0.05, Figure 9B). To find specific therapeutic agents suitable for either group, the IC50 ratio was set to >1.5 or <0.667 (displayed as |Log2(IC50_high-risk_/IC50_low-risk_)| > 0.585 in the plot), nine drugs were more sensitive in the high-risk group (gemcitabine, cytarabine, SCH772984, talazoparib, camptothecin, AZD6738, dasatinib, VX-11e and ERK-6604), and three drugs (TAF1-5496, AZD5991 and ABT737) in the low-risk group (Figure 9C; Supplementary Figures S7B–L). However, once the IC50 ratio was moved to 10 or 0.1 (|Log2(IC50_high-risk_/IC50_low-risk_)| >3.322), only gemcitabine, a commonly used chemotherapy agent, remained in the high-risk group preferentially (Figure 9C).

## 4 Discussion

The incidence rate of LC is increasing yearly and has currently become the second most common head and neck cancer (Bray et al., 2024). Due to its insidious onset, easy recurrence and treatment resistance, the 5-year overall survival rate of LC patients is only 25%–60% (Steuer et al., 2017). To improve survival, it is crucial to develop effective strategies for accurate prediction of the prognosis in LC patients and personalized therapies in clinics. Based on the functional role of UbRGs in the homeostasis of substrate proteins, various cellular processes are affected by their dysregulation. As suggested by accumulating evidence, tumourigenesis will be promoted in turn (Sun et al., 2020). On the other side, UbRGs had been employed as the marker genes for the prognosis of cancers. The predictive efficacy of the related signatures was reported as 69% in ovarian cancer and 65% in melanoma, respectively (Luo et al., 2023; Zhang et al., 2023). Therefore, systematic investigations of UbRGs are valuable to reveal their prognostic potential and oncological characteristics, and will possibly benefit the development of new applications of UbRGs in the prognosis and treatment of LC.

In this study, a total of 111 differentially expressed UbRGs were identified in LC and 3 of them, PPARG, LHX1 and LCK, were highlighted as the independent prognostic markers. The signature generated based on these three genes effectively discriminated LC patients with different OS and showed excellent applicability in most clinical conditions. The performance of this signature for 3-year OS in LC patients reached 81% and 70% in the training and validation sets, respectively, which was more powerful than earlier reported UbRGs-based signatures in other cancers (Luo et al., 2023; Zhang et al., 2023). The nomogram incorporating risk score and gender showed considerable advantages over other factors, such as the TNM stage and clinical stage. The consequent model had stronger predictive power and higher clinical benefit for 3-year OS in LC patients. In one word, this UbRGs-based signature will not only satisfy the risk stratification of LC patients but also enable the individualized assessment of the prognosis.

The functional linkages behind the UbRGs-based signature were then excavated with GSEA enrichment and subsequent panels of prediction, through which a significant association with immune in LC was demonstrated. T- and B-cell-mediated immune processes were enriched in the low-risk group, whereas there was no significant enrichment of immune processes in the high-risk group. As reported, UbRGs can induce the formation of immunosuppressive TME by affecting the stability of proteins important in the anti-tumor immune process, thereby promoting the immune escape of tumors (Çetin et al., 2021). For Instance, the E2 enzyme UBE2T inhibited CD8^+^ T-cell infiltration and expression of immune-promoting factors (IFN-γ, TNF-α and IL-2) in lung adenocarcinoma by activating the glycolytic pathway upon binding to FOXA1 (Pu et al., 2024). In colon cancer, a deubiquitinating enzyme USP4 suppressed anti-tumor immune responses by deubiquitinating TRAF6 and IRF3, hindering the nuclear localization of the latter protein and thus inhibiting cellular interferon responses and antigen presentation (Zhou et al., 2024). With further exploration of the immune microenvironment, it was observed that the low-risk group had a higher degree of infiltration of anti-tumor immune cells, more activated immune modules, stronger expression of immune-promoting cytokines and lower expression of immune-suppressing cytokines. Therefore, the active immune landscape in low-risk rather than high-risk populations may be one of the major forces shaping the different clinical outcomes.

Besides the correlation between cancer immunity and the entire signature in our study, reviewing the correlation with selected individual UbRGs also provides valuable insights into understanding the effectiveness of this signature. In previous reports, PPARG was mainly a regulator of immune cell differentiation and cytokine secretion (Riaz et al., 2023; Zhao et al., 2024). The essentiality of PPARG was indicated in the differentiation of fetal monocytes into alveolar macrophages (Schneider et al., 2014). In mouse models of colitis, it was observed that PPARG agonists can shift the immune response from a Th1-type to a Th2-type, resulting in a decrease in the expression of Th1-associated transcription factors, cytokines, and chemokines, and simultaneously an increase in the expression of Th2-associated factors (Celinski et al., 2013; Saubermann et al., 2002). Deficiency of this gene in a similar mouse model was associated with a decreased number of CD4^+^Foxp3^+^ regulatory T cells (Guri et al., 2010). Additionally, PPARG was also observed to inhibit the secretion of pro-inflammatory cytokines (such as TNF-α and IL-1β) and promote anti-inflammatory cytokines (such as TGF-β and IL-10) (Riaz et al., 2023). In a cancerous context, Liu *et al* reported the accelerative role played by activated PPARG in KRAS-mutant pancreatic carcinogenesis. The tumor immune microenvironment was remodeled by PPARG via recruiting and promoting the M2 polarization of macrophages through the CCL2/CCR2 signaling axis (Liu et al., 2022). But the actual functional roles of PPARG playing in laryngeal cancer are still lack of investigation. Similar to our work, the other risk gene LHX1 was also adopted in a recent published prognostic signature of breast cancer. With consistence, correlation was observed between LHX1 and lymph node metastasis, infiltration of multiple immune cells (including CD8^+^ T cells, B cells, dendritic cells, antigen-presenting cells, neutrophils and regulatory T cells) and enrichment of immune functions in patients (including B-cell receptor signaling pathway, PD-L1 expression, and the PD-1 checkpoint pathway) (Pan et al., 2024). However, the potential role of LHX1 was only suggested with the trend of dysregulation in cancer, but not yet by the functional assays in normal and oncogenic circumstance. Additionally, the protective gene LCK, one of the non-receptor tyrosine kinases in the Src family, was reported as a crucial player in T cell-mediated immune responses in previous reports (Wu et al., 2021; Lanz et al., 2024). It precisely regulated T cell activation and the subsequent cascade of immune reactions by initiating T cell receptor (TCR) signal transduction. Once TCR binding to the antigenic peptide-MHC complex, LCK was activated with the synergistic participation of co-receptors CD4 or CD8. The activated LCK prompted the subsequent phosphorylation of CD3 and ζ-chain immunoreceptor tyrosine-based activation motifs, recruiting and activating ZAP-70, and led to the formation of LAT signaling bodies through further phosphorylation of LAT and SLP-76. A panel of downstream signaling pathways were triggered consequently, including ERK and PI3K/Akt, and thus, the T cell-mediated immune responses were launched ultimately. Meanwhile, LCK can also indirectly connect ZAP-70 and LAT, and promote their phosphorylation, thus TCR signal transduction is enhanced (De Sanctis et al., 2024). Another report about Jurkat E6-1 leukemia cells by Wan *et al* demonstrated the expression of LCK was under the regulation of SMAD4, and affected the proliferation of chimeric antigen receptor-T cells through perturbation of PI3K/Akt signal (Wan et al., 2025). In multiple tumor cell lines, as reported by Ahn *et al*, the tumor surveillance was mediated by LCK-ERK signal through the activation of T cells (Ahn et al., 2025). These accumulating clues suggested the rationality of our choice of signature genes, and further, the derived risk signature for prognosis.

In our wet-lab works, the expression was validated for 12 cytokines predicted according to the according to the association with signature genes, either promotors and suppressors of cancer immunity. Among them, four immunosuppressors supported by PPARG were confirmed through CRISPR-based gene knockout, including IL6, TGFB1, TGFB2 and VEGFC, which was consistent with an earlier report (Riaz et al., 2023). Since then, the cytokine expression and functions in LCs are worthy of further investigation to provide more insights into our signature and help the stratification of patients.

Another potential value of this signature is to facilitate the optimization of clinical treatments for LC patients. Based on the properties of the immune microenvironment, the low-risk group tends to be “hot” tumors, while the high-risk group tends to be “cold” (Duan et al., 2020). Due to the presence of higher numbers of effector T cells in “hot” tumors, combined with the preferential expression of 10 checkpoint genes, treatment with immune checkpoint inhibitors will be more effective in the low-risk group (Cejuela et al., 2022). Of all the targets that predominate in the low-risk group, immune checkpoint inhibitors against PDCD1, CD27, CD40LG, BTLA and LAG3 have been approved for clinical use or trials in patients with other tumors (Sharma et al., 2024; Lutfi et al., 2021; Liu et al., 2021; Dalle et al., 2024; Ibrahim et al., 2023), and thus, worthy to be tried in LC patients. Additionally, the prediction of drug sensitivity showed little preference between the high- and low-risk groups scored with the UbRGs-based signature, except for gemcitabine, a traditional chemotherapy agent, which showed hypersensitivity in the high-risk group. This drug works through inhibition of DNA synthesis as pyrimidine antimetabolites and is commonly applied in pancreatic cancer but not LC (Han et al., 2022). However, based on our signature, at least a certain portion of LC patients in the high-risk group may benefit from the administration of gemcitabine, which will be a potential alternative choice for LC patients, like cisplatin and paclitaxel (Fang et al., 2023). Regardless, the UbRGs-based signature provides new insights into the choice of therapeutic agents and strategies for LC.

Despite the encouraging performance and advantages of the UbRGs-based prognostic signature, more investigations shall be carried out in the future. Firstly, due to the limited number of LC patients contained in the TCGA and GEO databases, larger clinical cohorts are necessary for comprehensive validation of this signature. Secondly, the oncological and immunological roles of PPARG, LHX1 and LCK should be explored in depth, particularly in LC, to specify their functions and prognostic values. Thirdly, the prediction of drug sensitivity based on the signature still requires extensive assessments in different models and ultimately in patients, since the prediction was fundamentally based on the collection of gene expression profiles in cancer cell lines.

## 5 Conclusion

In conclusion, we systematically analyzed the molecular characteristics and prognostic potential of UbRGs in LC for the first time, and established a prognostic signature based on UbRGs. This signature demonstrated good clinical value in predicting the patients’ prognosis, speculating the immune microenvironment and suggesting anticancer therapies, thus facilitating the risk stratification of clinical patients and providing new ideas for formulating individualized treatment.

## Data Availability

The datasets presented in this study can be found in online repositories. The names of the repository/repositories and accession number(s) can be found in the article/Supplementary Material.

## References

[B1] Ahn J. Jang S. H. Jang S. Yoon J. H. Lee M. G. Chi S. G. (2025). XAF1 is secreted from stressed tumor cells to activate T cell-mediated tumor surveillance via Lck-ERK signaling. Neoplasia 59, 101094. 10.1016/j.neo.2024.101094 39615106 PMC11646784

[B2] Balachandran V. P. Gonen M. Smith J. J. DeMatteo R. P. (2015). Nomograms in oncology: more than meets the eye. Lancet Oncol. 16 (4), e173–e180. 10.1016/S1470-2045(14)71116-7 25846097 PMC4465353

[B3] Bray F. Laversanne M. Sung H. Ferlay J. Siegel R. L. Soerjomataram I. (2024). Global cancer statistics 2022: GLOBOCAN estimates of incidence and mortality worldwide for 36 cancers in 185 countries. CA Cancer J. Clin. 74 (3), 229–263. 10.3322/caac.21834 38572751

[B4] Cejuela M. Vethencourt A. Pernas S. (2022). Immune checkpoint inhibitors and novel immunotherapy approaches for breast cancer. Curr. Oncol. Rep. 24 (12), 1801–1819. 10.1007/s11912-022-01339-4 36255603

[B5] Celinski K. Dworzanski T. Fornal R. Korolczuk A. Madro A. Brzozowski T. (2013). Comparison of anti-inflammatory properties of peroxisome proliferator-activated receptor gamma agonists rosiglitazone and troglitazone in prophylactic treatment of experimental colitis. J. Physiol. Pharmacol. 64 (5), 587–595. 24304573

[B6] Çetin G. Klafack S. Studencka-Turski M. Krüger E. Ebstein F. (2021). The ubiquitin-proteasome system in immune cells. Biomolecules 11 (1), 60. 10.3390/biom11010060 33466553 PMC7824874

[B7] Cui J. Wang L. Tan G. Chen W. He G. Huang H. (2020a). Development and validation of nomograms to accurately predict risk of recurrence for patients with laryngeal squamous cell carcinoma: cohort study. Int. J. Surg. 76, 163–170. 10.1016/j.ijsu.2020.03.010 32173614

[B8] Cui J. Wang L. Zhong W. Chen Z. Tan X. Yang H. (2020b). Development and validation of nomogram to predict risk of survival in patients with laryngeal squamous cell carcinoma. Biosci. Rep. 40 (8), BSR20200228. 10.1042/BSR20200228 32744320 PMC7432998

[B9] Dai W. L. Yuan S. X. Cao J. P. (2020). The deubiquitinase USP34 stabilizes SOX2 and induces cell survival and drug resistance in lar-yngeal squamous cell carcinoma. Kaohsiung J. Med. Sci. 36 (12), 983–989. 10.1002/kjm2.12285 32783291 PMC11896225

[B10] Dalle S. Verronese E. N'Kodia A. Bardin C. Rodriguez C. Andrieu T. (2024). Modulation of blood T cell polyfunctionality and HVEM/BTLA expression are critical determinants of clinical outcome in anti-PD1-treated metastatic melanoma patients. Oncoimmunology 13 (1), 2372118. 10.1080/2162402X.2024.2372118 38939518 PMC11210932

[B11] De Sanctis J. B. Garmendia J. V. Duchová H. Valentini V. Puskasu A. Kubíčková A. (2024). Lck function and modulation: immune cytotoxic response and tumor treatment more than a simple event. Cancers 16 (15), 2630. 10.3390/cancers16152630 39123358 PMC11311849

[B12] Díaz P. Sandoval-Bórquez A. Bravo-Sagua R. Quest A. F. G. Lavandero S. (2021). Perspectives on organelle interaction, protein dysregulation, and cancer disease. Front. Cell Dev. Biol. 9, 613336. 10.3389/fcell.2021.613336 33718356 PMC7946981

[B13] Duan Q. Zhang H. Zheng J. Zhang L. (2020). Turning cold into hot: firing up the tumor microenvironment. Trends Cancer 6 (7), 605–618. 10.1016/j.trecan.2020.02.022 32610070

[B14] Egelmeer A. G. Velazquez E. R. De Jong J. M. Oberije C. Geussens Y. Nuyts S. (2011). Development and validation of a nomogram for prediction of survival and local control in laryngeal carcinoma patients treated with radiotherapy alone: a cohort study based on 994 patients. Radiother. Oncol. 100 (1), 108–115. 10.1016/j.radonc.2011.06.023 21784544

[B15] Fang Q. Xu P. Cao F. Wu D. Liu X. (2023). PD-1 Inhibitors combined with paclitaxel (Albumin-bound) and cisplatin for larynx preservation in locally advanced laryngeal and hypopharyngeal squamous cell carcinoma: a retrospective study. Cancer Immunol. Immunother. 72 (12), 4161–4168. 10.1007/s00262-023-03550-z 37804437 PMC10992232

[B16] Guri A. J. Mohapatra S. K. Horne W. T. 2nd Hontecillas R. Bassaganya-Riera J. (2010). The role of T cell PPAR gamma in mice with experimental inflammatory bowel disease. BMC Gastroenterol. 10, 60. 10.1186/1471-230X-10-60 20537136 PMC2891618

[B17] Han H. Li S. Zhong Y. Huang Y. Wang K. Jin Q. (2022). Emerging pro-drug and nano-drug strategies for gemcita-bine-based cancer therapy. Asian J. Pharm. Sci. 17 (1), 35–52. 10.1016/j.ajps.2021.06.001 35261643 PMC8888143

[B18] Ibrahim R. Saleh K. Chahine C. Khoury R. Khalife N. Cesne A. L. (2023). LAG-3 inhibitors: novel immune checkpoint inhibitors changing the landscape of immunotherapy. Biomedicines 11 (7), 1878. 10.3390/biomedicines11071878 37509517 PMC10377063

[B19] Lanz A. L. Erdem S. Ozcan A. Ceylaner G. Cansever M. Ceylaner S. (2024). A novel biallelic LCK variant resulting in profound T-cell immune deficiency and review of the literature. J. Clin. Immunol. 44 (1), 1. 10.1007/s10875-023-01602-8 PMC10724324 38100037

[B20] Liang M. Sun Z. Chen X. Wang L. Wang H. Qin L. (2023). E3 ligase TRIM28 promotes anti-PD-1 resistance in non-small cell lung cancer by enhancing the recruitment of myeloid-derived suppressor cells. J. Exp. Clin. Cancer Res. 42 (1), 275. 10.1186/s13046-023-02862-3 37865804 PMC10589970

[B21] Liu Y. Deguchi Y. Wei D. Liu F. Moussalli M. J. Deguchi E. (2022). Rapid acceleration of KRAS-mutant pancreatic carcinogenesis via remodeling of tumor immune microenvironment by PPARδ. Nat. Commun. 13 (1), 2665. 10.1038/s41467-022-30392-7 35562376 PMC9106716

[B22] Liu Z. Liu L. Guo C. Yu S. Meng L. Zhou X. (2021). Tumor suppressor gene mutations correlate with prognosis and im-munotherapy benefit in hepatocellular carcinoma. Int. Immunopharmacol. 101 (Pt B), 108340. 10.1016/j.intimp.2021.108340 34789428

[B23] Luo X. Wang Y. Zhang H. Chen G. Sheng J. Tian X. (2023). Identification of a prognostic signature for ovarian cancer based on ubiquitin-related genes suggesting a potential role for FBXO9. Biomolecules 13 (12), 1724. 10.3390/biom13121724 38136595 PMC10742228

[B24] Lutfi F. Wu L. Sunshine S. Cao X. (2021). Targeting the CD27-CD70 pathway to improve outcomes in both checkpoint immuno-therapy and allogeneic hematopoietic cell transplantation. Front. Immunol. 12, 715909. 10.3389/fimmu.2021.715909 34630390 PMC8493876

[B25] Pan Y. Zou Q. Yin W. Huang Z. Zhao Y. Mo Z. (2024). Development of lymph node metastasis-related prognostic markers in breast cancer. J. Proteomics 291, 105045. 10.1016/j.jprot.2023.105045 37939914

[B26] Pu J. Zhang D. Wang B. Zhu P. Yang W. Wang K. (2024). FOXA1/UBE2T inhibits CD8+T cell activity by inducing mediates glycolysis in lung adenocarcinoma. Front. Biosci. Landmark Ed. 29 (4), 134. 10.31083/j.fbl2904134 38682180

[B27] Riaz F. Wei P. Pan F. (2023). PPARs at the crossroads of T cell differentiation and type 1 diabetes. Front. Immunol. 14, 1292238. 10.3389/fimmu.2023.1292238 37928539 PMC10623333

[B28] Saubermann L. J. Nakajima A. Wada K. Zhao S. Terauchi Y. Kadowaki T. (2002). Peroxisome proliferator-activated receptor gamma agonist ligands stimulate a Th2 cytokine response and prevent acute colitis. Inflamm. Bowel Dis. 8 (5), 330–339. 10.1097/00054725-200209000-00004 12479648

[B29] Schneider C. Nobs S. P. Kurrer M. Rehrauer H. Thiele C. Kopf M. (2014). Induction of the nuclear receptor PPAR-γ by the cytokine GM-CSF is critical for the differentiation of fetal monocytes into alveolar macrophages. Nat. Immunol. 15 (11), 1026–1037. 10.1038/ni.3005 25263125

[B30] Sharma N. Mazumder R. Rai P. Debnath A. (2024). Role of PD-1 in skin cancer: molecular mechanism, clinical applications, and resistance. Chem. Biol. Drug Des. 104 (3), e14613. 10.1111/cbdd.14613 39231792

[B31] Shi D. Wu X. Jian Y. Wang J. Huang C. Mo S. (2022). USP14 promotes tryptophan metabolism and immune suppression by stabilizing Ido1 in colorectal cancer. Nat. Commun. 13 (1), 5644. 10.1038/s41467-022-33285-x 36163134 PMC9513055

[B32] Steuer C. E. El-Deiry M. Parks J. R. Higgins K. A. Saba N. F. (2017). An update on larynx cancer. CA Cancer J. Clin. 67 (1), 31–50. 10.3322/caac.21386 27898173

[B33] Sun T. Liu Z. Yang Q. (2020). The role of ubiquitination and deubiquitination in cancer metabolism. Mol. Cancer 19 (1), 146. 10.1186/s12943-020-01262-x 33004065 PMC7529510

[B34] Wan R. Fu B. Fu X. Liu Z. Simayi N. Fu Y. (2025). SMAD4 regulates the expression of LCK affecting chimeric antigen receptor-T cells proliferation through PI3K/akt signaling pathway. J. Cell Physiol. 240 (1), e31520. 10.1002/jcp.31520 39763264 PMC11704455

[B35] Wang K. Tang J. Liu X. Wang Y. Chen W. Zheng R. (2020). UBR5 regulates proliferation and radiosensitivity in human laryngeal carcinoma via the p38/MAPK signaling pathway. Oncol. Rep. 44 (2), 685–697. 10.3892/or.2020.7620 32468011 PMC7336417

[B36] Wu J. Li G. Li L. Li D. Dong Z. Jiang P. (2021). Asparagine enhances LCK signalling to potentiate CD8+ T-cell activation and an-ti-tumour responses. Nat. Cell Biol. 23 (1), 75–86. 10.1038/s41556-020-00615-4 33420490

[B37] Zhang L. Shi Z. Zhang F. Chen B. Qiu W. Cai L. (2023). Ubiquitination-related biomarkers in metastatic melanoma patients and their roles in tumor microenvironment. Front. Oncol. 13, 1170190. 10.3389/fonc.2023.1170190 37274231 PMC10235493

[B38] Zhao Y. Tan H. Zhang X. Zhu J. (2024). Roles of peroxisome proliferator-activated receptors in hepatocellular carcinoma. J. Cell Mol. Med. 28 (5), e18042. 10.1111/jcmm.18042 37987033 PMC10902579

[B39] Zhou Y. Li H. Zhang Y. Zhao E. Huang C. Pan X. (2024). Deubiquitinase USP4 suppresses an-titumor immunity by inhibiting IRF3 activation and tumor cell-intrinsic interferon response in colorectal cancer. Cancer Lett. 589, 216836. 10.1016/j.canlet.2024.216836 38556105

