# Supplementary figures and images for: A novel ubiquitin-related genes-based signature demonstrated values in prognostic prediction, immune landscape sculpture and therapeutic options in laryngeal cancer
Source: Front Pharmacol. 2025 Mar 20;16:1513948. doi: 10.3389/fphar.2025.1513948 (PMC11965687; doi:10.3389/fphar.2025.1513948)

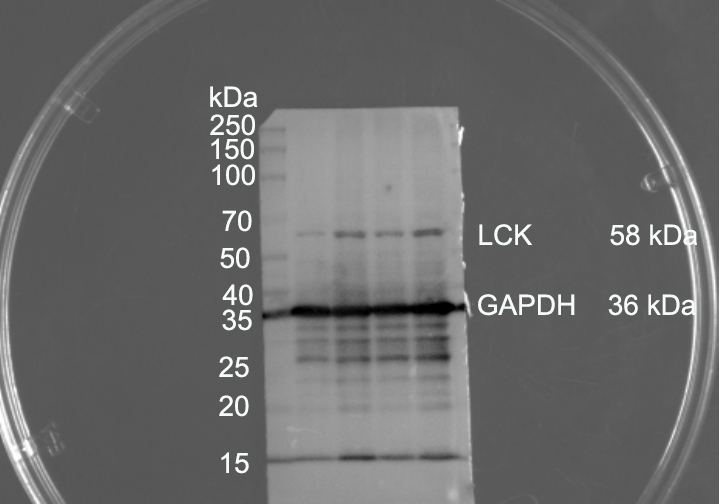

Supplement: Supplementary file 1 [file Presentation1.zip › 5.Raw data of western blots/LCK/GAPDH.jpg]

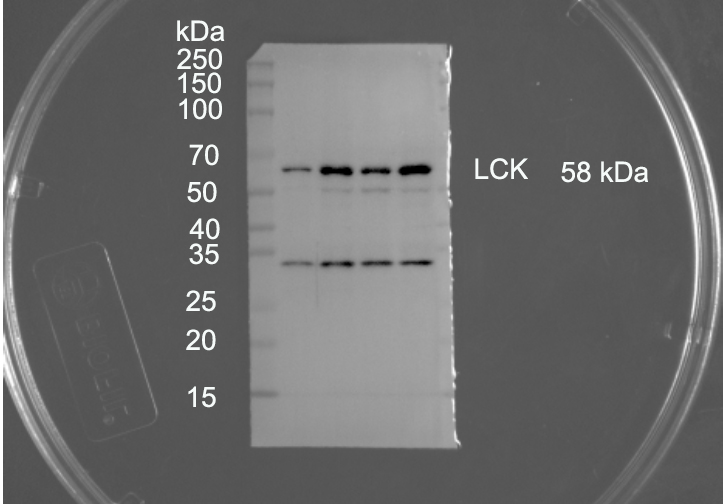

Supplement: Supplementary file 1 [file Presentation1.zip › 5.Raw data of western blots/LCK/LCK.jpg]

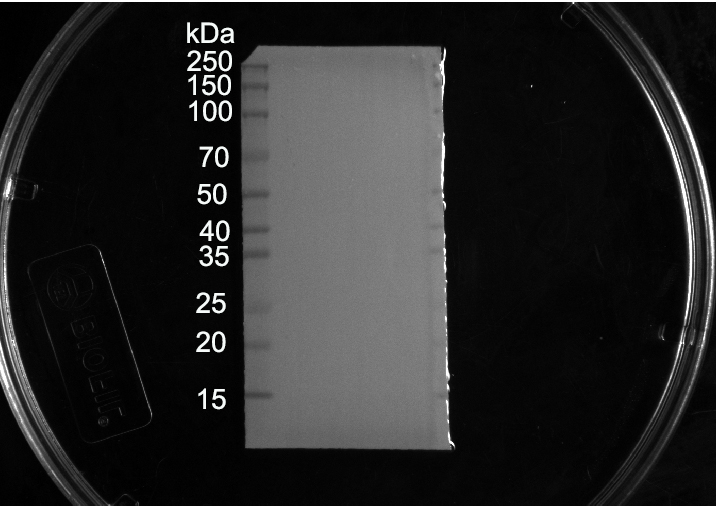

Supplement: Supplementary file 1 [file Presentation1.zip › 5.Raw data of western blots/LCK/Maker.jpg]

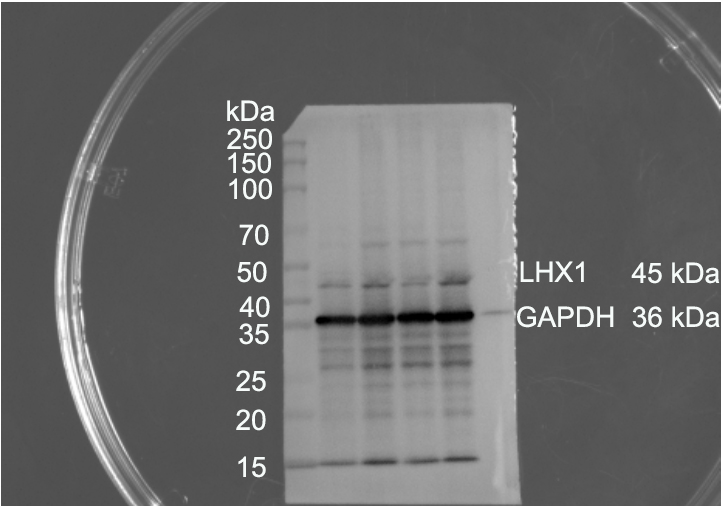

Supplement: Supplementary file 1 [file Presentation1.zip › 5.Raw data of western blots/LHX1/GAPDH.jpg]

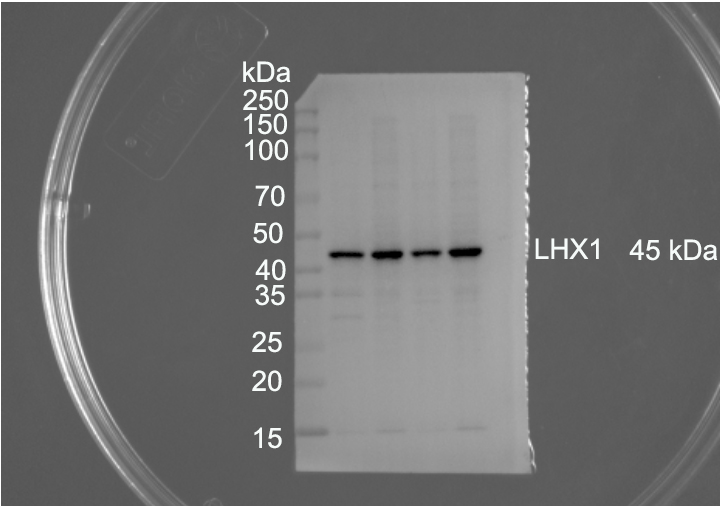

Supplement: Supplementary file 1 [file Presentation1.zip › 5.Raw data of western blots/LHX1/LHX1.jpg]

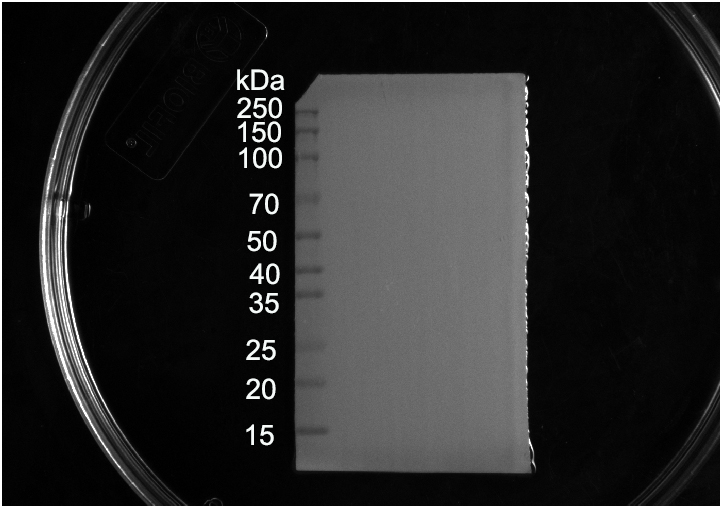

Supplement: Supplementary file 1 [file Presentation1.zip › 5.Raw data of western blots/LHX1/Maker.jpg]

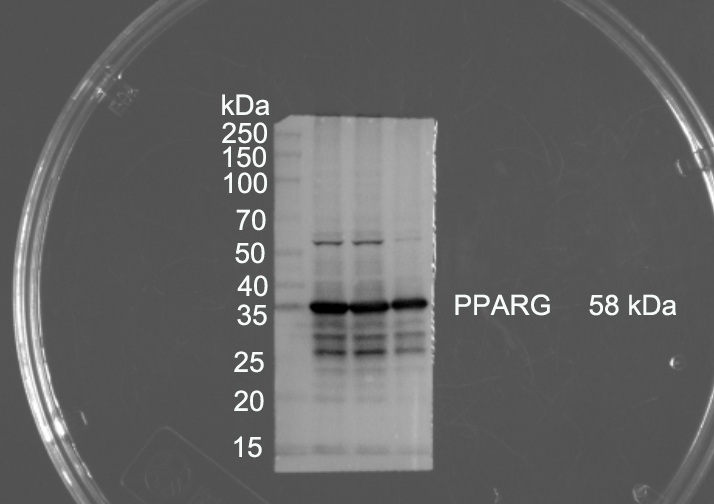

Supplement: Supplementary file 1 [file Presentation1.zip › 5.Raw data of western blots/PPARG-KO-TU212/GAPDH.jpg]

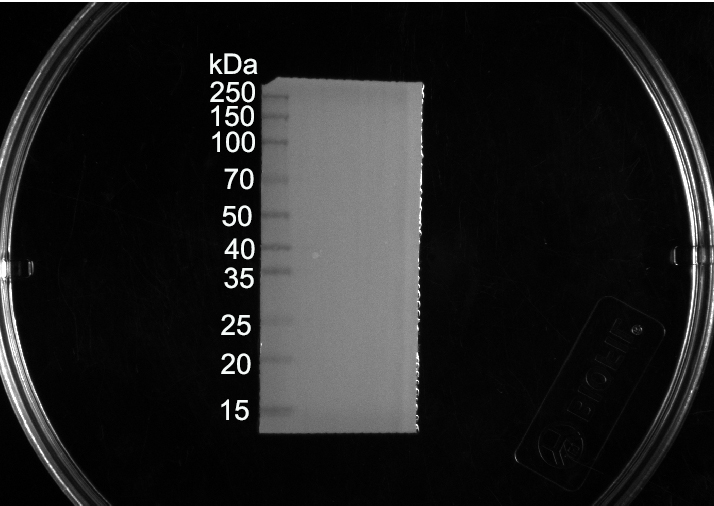

Supplement: Supplementary file 1 [file Presentation1.zip › 5.Raw data of western blots/PPARG-KO-TU212/Maker.jpg]

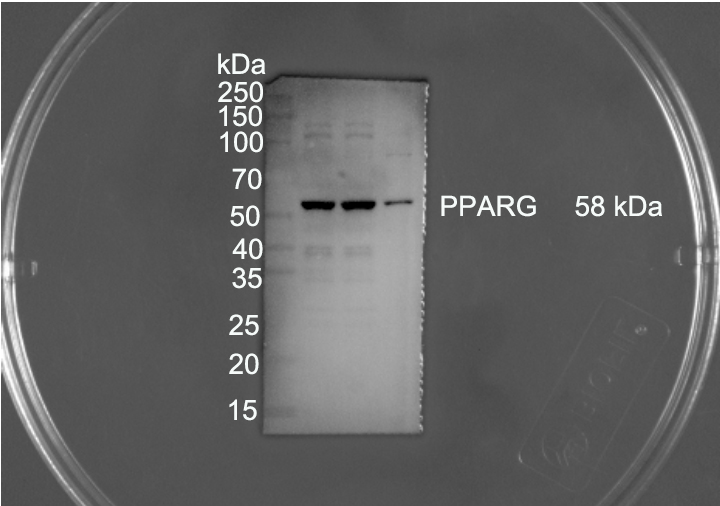

Supplement: Supplementary file 1 [file Presentation1.zip › 5.Raw data of western blots/PPARG-KO-TU212/PPARG.jpg]

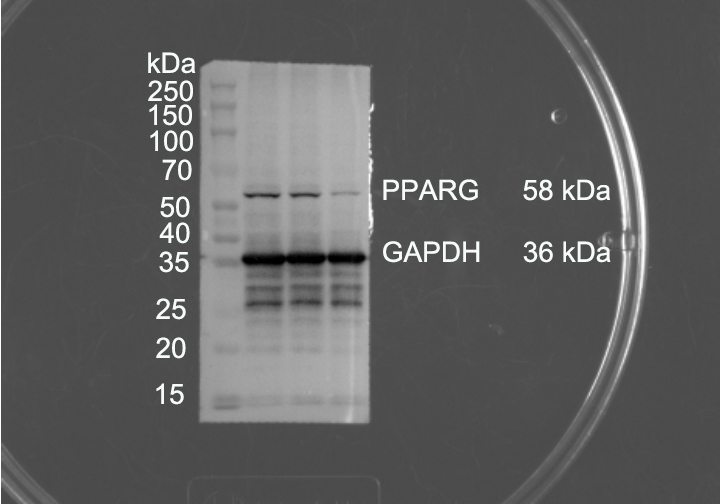

Supplement: Supplementary file 1 [file Presentation1.zip › 5.Raw data of western blots/PPARG-KO-TU686/GAPDH.jpg]

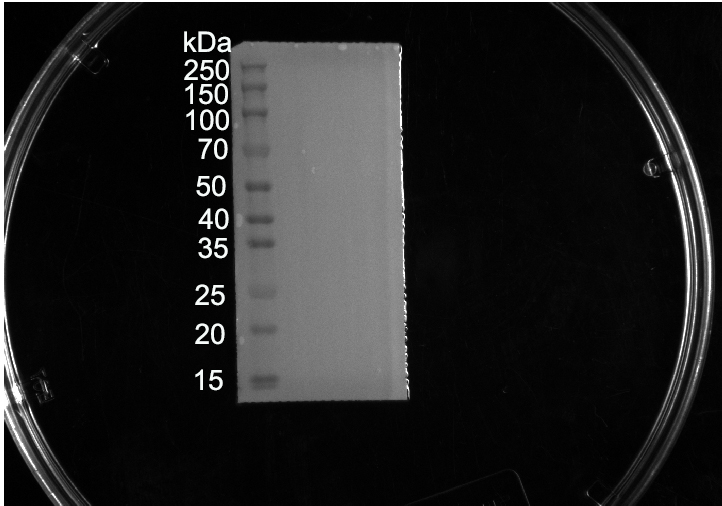

Supplement: Supplementary file 1 [file Presentation1.zip › 5.Raw data of western blots/PPARG-KO-TU686/Maker.jpg]

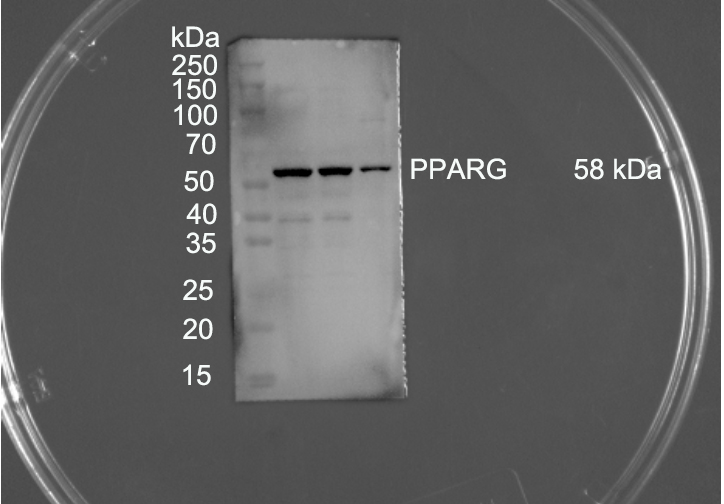

Supplement: Supplementary file 1 [file Presentation1.zip › 5.Raw data of western blots/PPARG-KO-TU686/PPARG.jpg]

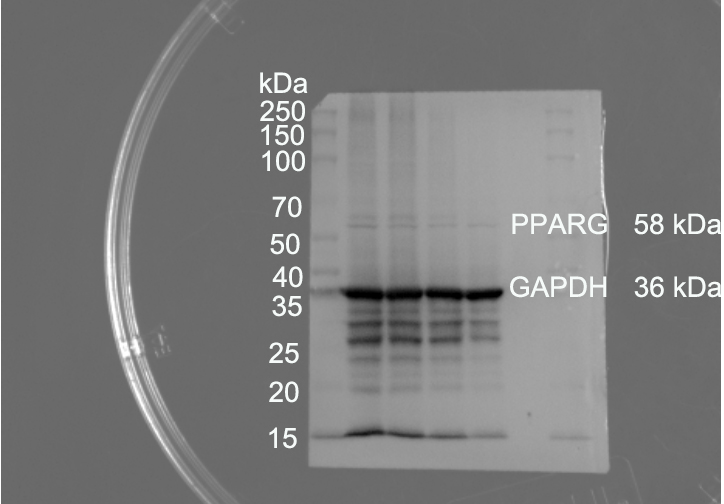

Supplement: Supplementary file 1 [file Presentation1.zip › 5.Raw data of western blots/PPARG/GAPDH.jpg]

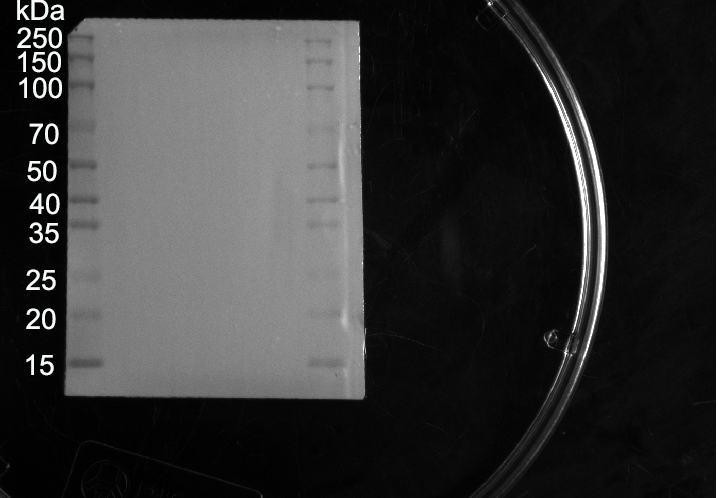

Supplement: Supplementary file 1 [file Presentation1.zip › 5.Raw data of western blots/PPARG/Maker.jpg]

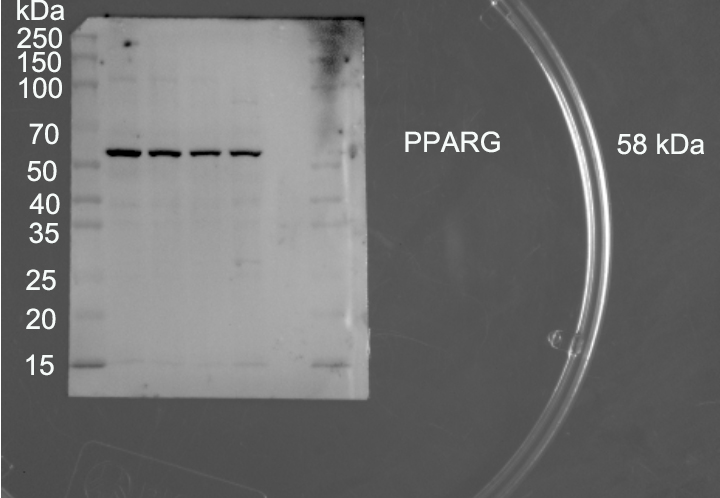

Supplement: Supplementary file 1 [file Presentation1.zip › 5.Raw data of western blots/PPARG/PPARG.jpg]
