# Supplementary material for: A novel ubiquitin-related genes-based signature demonstrated values in prognostic prediction, immune landscape sculpture and therapeutic options in laryngeal cancer
Source: Front Pharmacol. 2025 Mar 20;16:1513948. doi: 10.3389/fphar.2025.1513948 (PMC11965687; doi:10.3389/fphar.2025.1513948)
Supplement: Supplementary file 2 [file DataSheet1.pdf]

Supplementary Material

1 Supplementary Data: uploaded in a separate ZIP file, including:

1.1 Training and validation datasets of LC patients.

1.2 Excel list of UbRGs.

1.3 Excel list of GSEA results.

1.4 Source datasets for evaluation of drug sensitivity.

1.5 Raw data of western blots.

2 Supplementary Figures and Tables

2.1 Supplementary Figure 1. Functional analyses of DUbRGs.

2.2 Supplementary Figure 2. Validation of the 3 signature genes.

2.3 Supplementary Figure 3. Kaplan-Meier analysis of OS in stratified conditions.

2.4 Supplementary Figure 4. Proportions of enriched items of GO and KEGG.

2.5 Supplementary Figure 5. GSEA plots of high-risk group and the immune landscape.

2.6 Supplementary Figure 6. Expression of cytokines in PPARG knockout LC cells.

2.7 Supplementary Figure 7. Prediction of sensitivity to clinical therapeutic agents.

2.8 Supplementary Table 1. The weblinks of databases and online tools used in this study.

2.9 Supplementary Table 2. Patient number and statistical significance of survival analysis.

2.10 Supplementary Table 3. sgRNA sequences for control and PPARG knockout.

2.11 Supplementary Table 4. Primer sequences used for qRT-PCR.

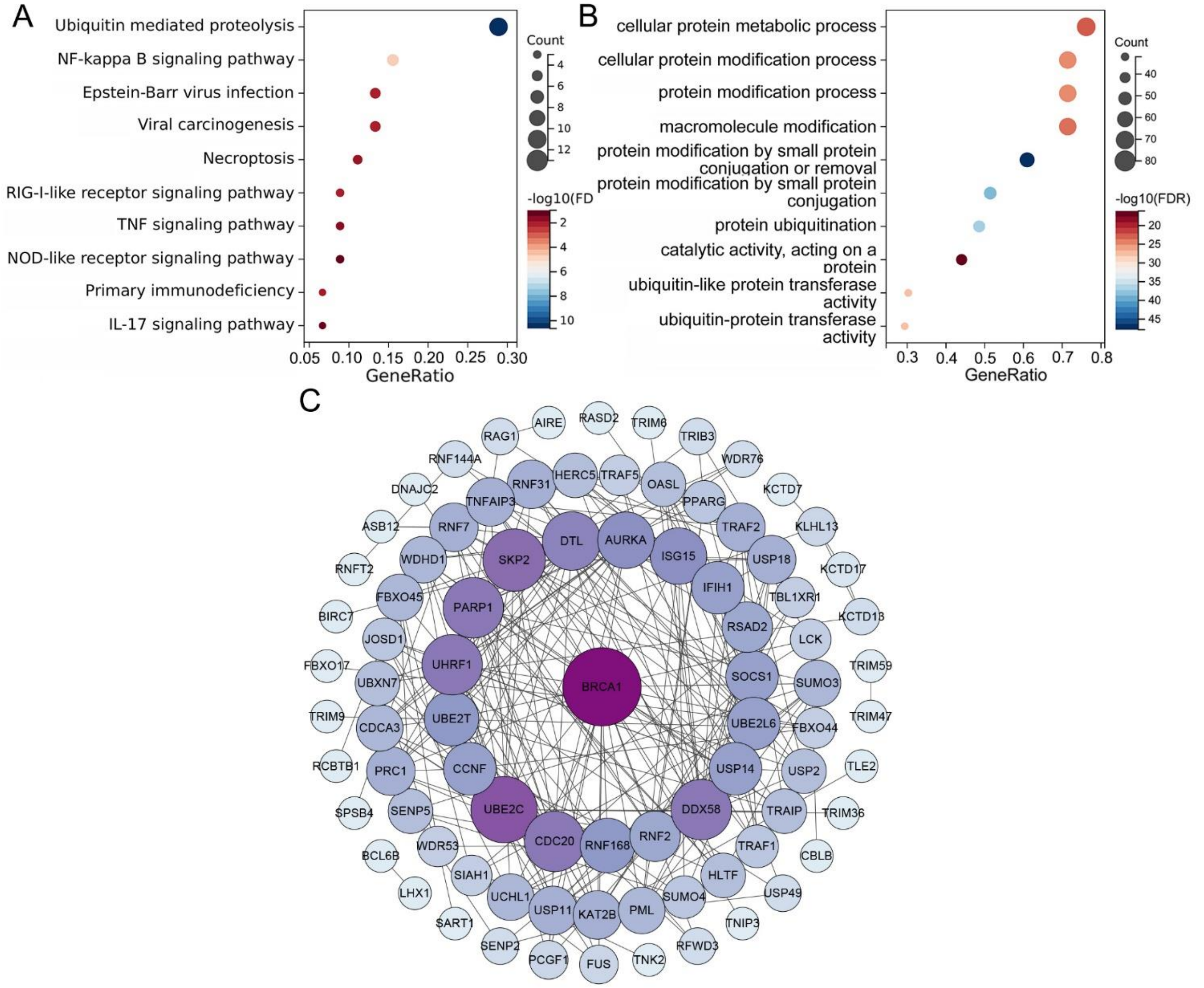

Supplementary Figure 1. Functional analyses of DUbRGs. (A, B) The top 10 pathways enriched in DUbRGs were presented based on (A) KEGG and (B) GO. (C) The PPI network of DUbRGs.

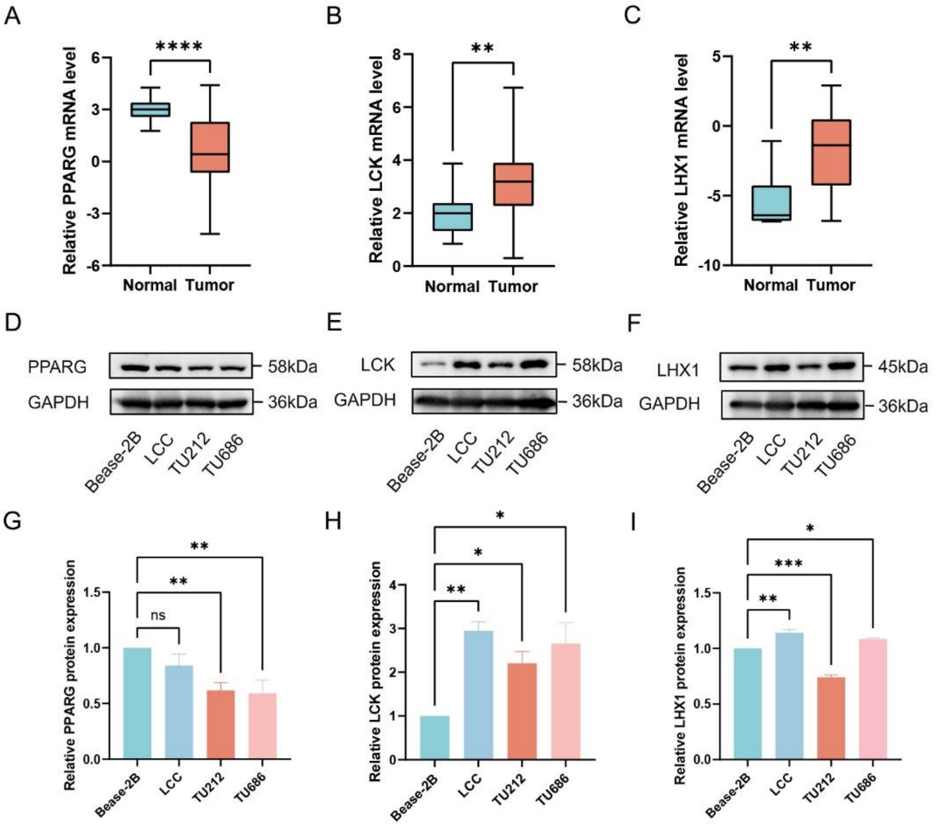

Supplementary Figure 2. Validation of the 3 signature genes. (A-C) Box plots of mRNA levels of signature genes in normal and LC patients retrieved from the training set, (A) PPARG, (B) LCK and (C) LHX1. (D-F) Protein levels of signature genes in 1 normal (Bease-2B) and 3 LC cell lines (LCC, TU212 and TU686) were assessed with western blot, (D) PPARG, (E) LCK and (F) LHX1. (G-I) Quantification of protein levels based on western blot results, (G) PPARG, (H) LCK and (I) LHX1. * $p < 0.05$; ** $p < 0.01$; *** $p < 0.001$; **** $p < 0.0001$.

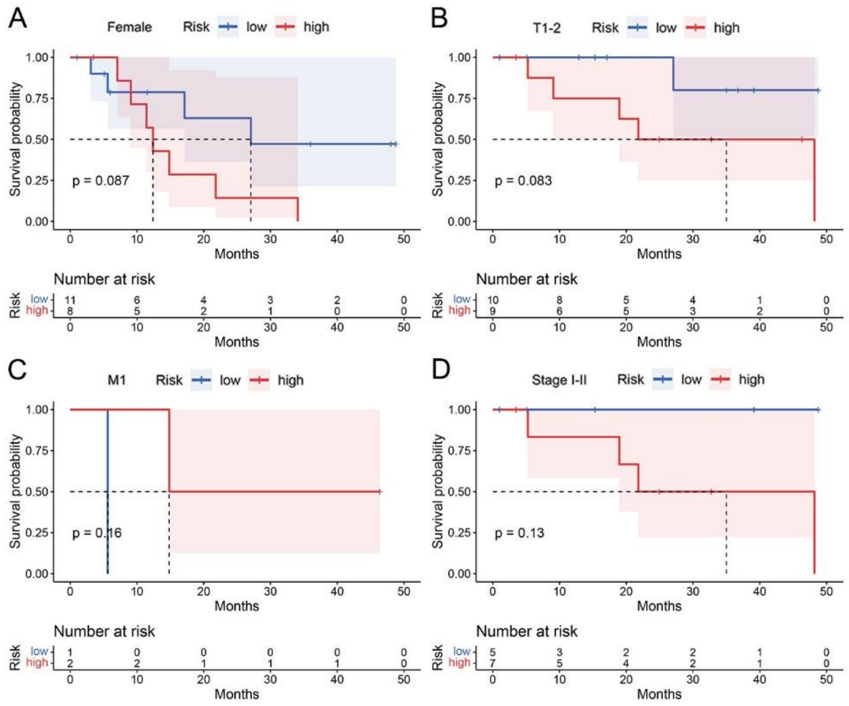

Supplementary Figure 3. Kaplan-Meier analysis of OS in stratified conditions. (A) Female, (B) T1-2 stage, (C) M1 stage and (D) clinical stage I-II showed no significant differences in OS between the high- and low-risk groups.

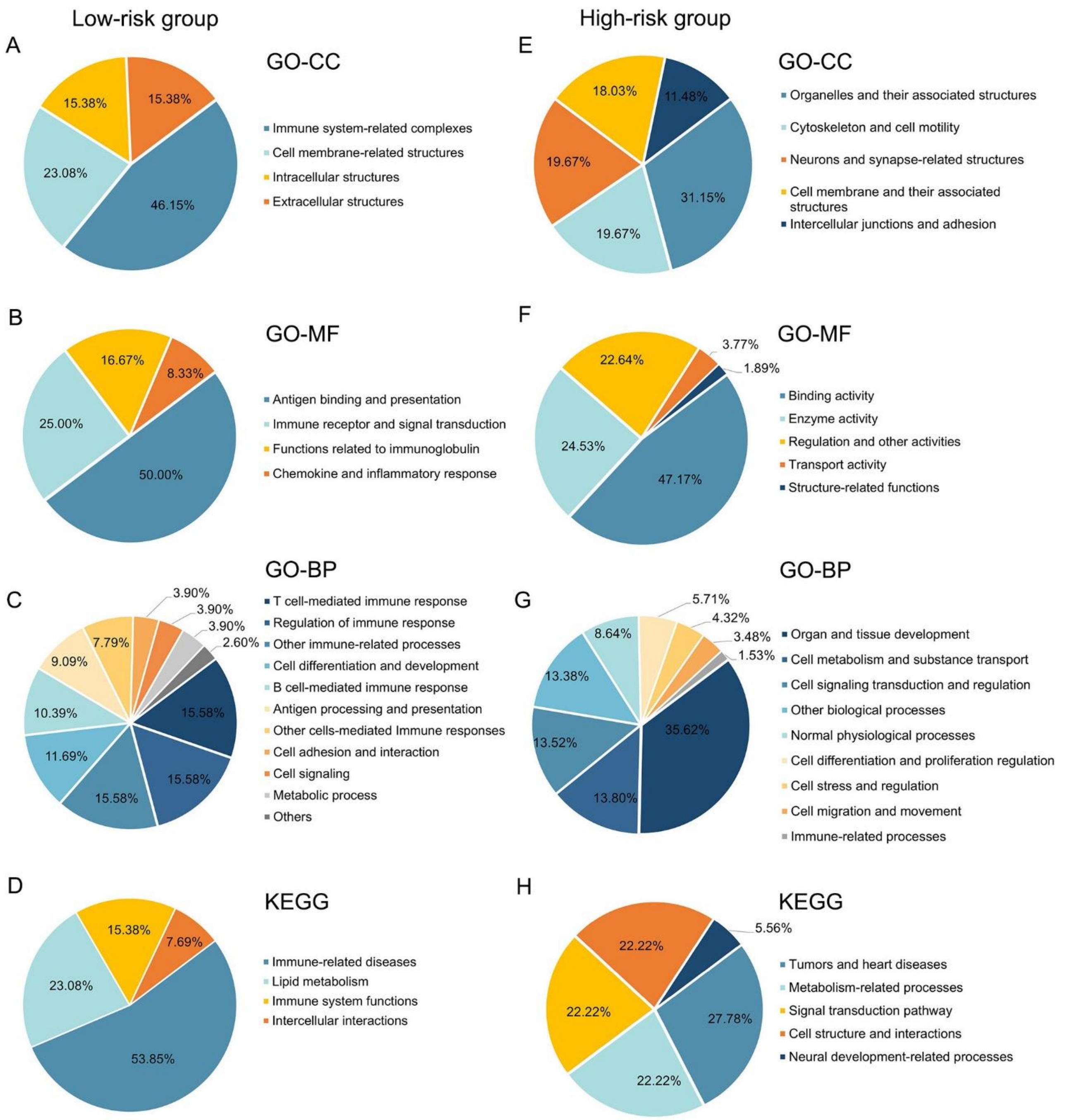

Supplementary Figure 4. Proportions of enriched items of GO and KEGG. (A-D) Items enriched in the low-risk group were presented in the left column, whereas (E-F) items enriched with high-risk at the right side. (A, E) GO-Cellular Component (CC), (B, F) Molecular Function (MF), (C, G) Biological Process (BP) and (D, H) KEGG.

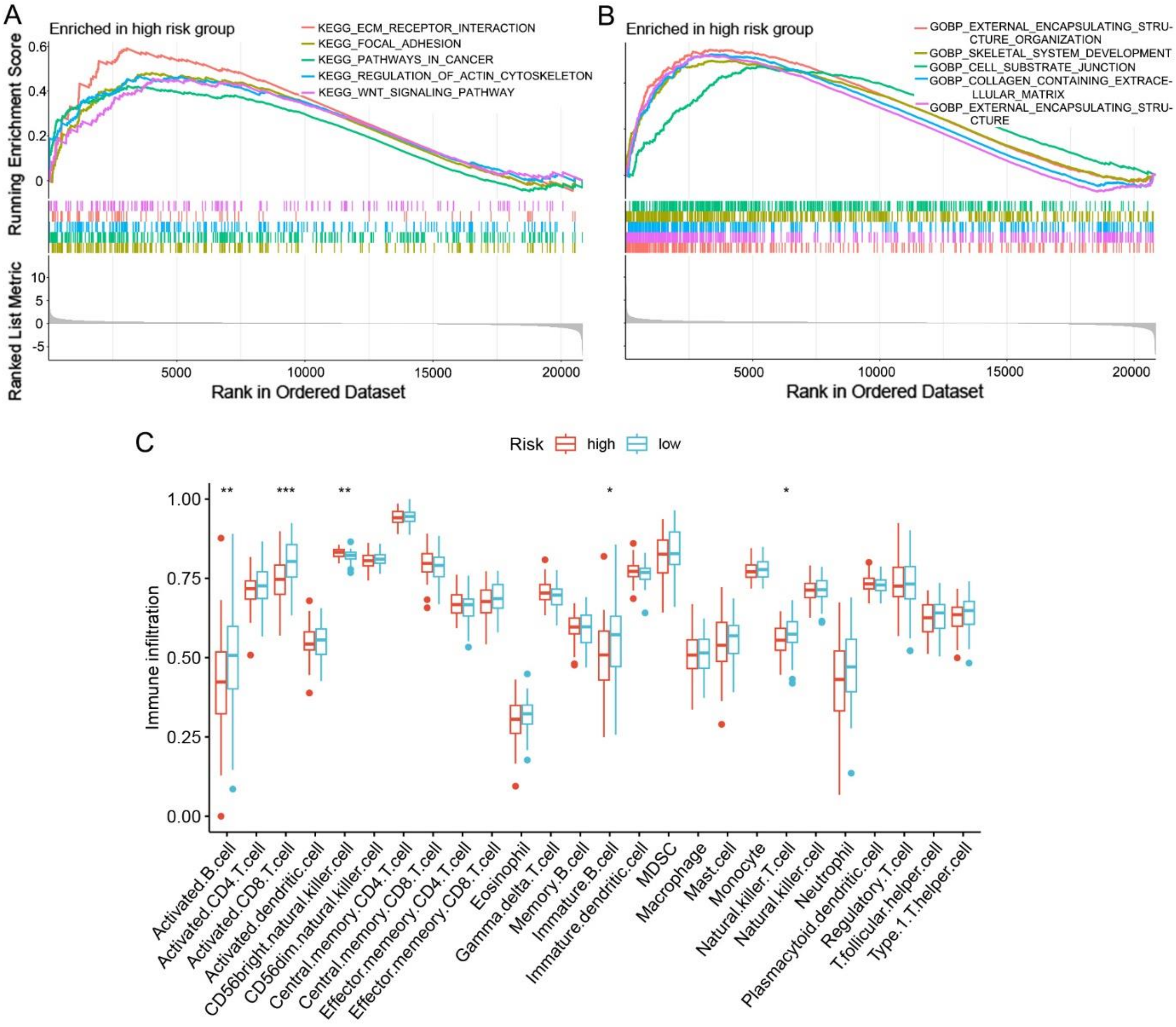

Supplementary Figure 5. GSEA plots of high-risk group and the immune landscape. (A, B) GSEA enrichment analysis in the high-risk group based on KEGG- (A) and Go- (B) related gene sets. (C) Immune cell infiltration was analyzed with the ssGSEA algorithm and 5 cell types were highlighted with statistical significance between the high- and low-risk groups. * $p < 0.05$; ** $p < 0.01$; *** $p < 0.001$.

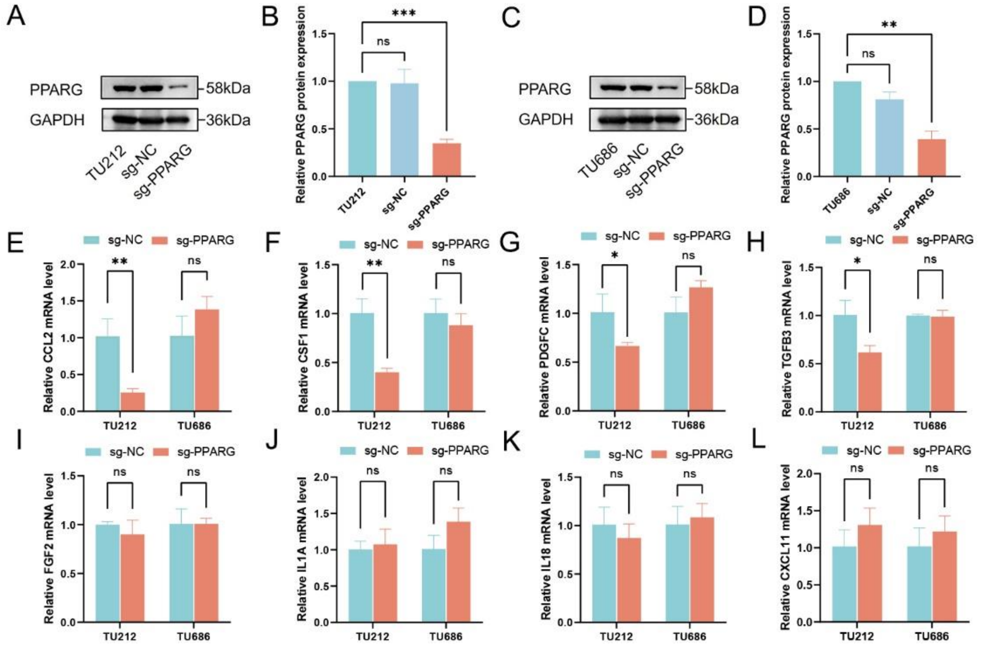

Supplementary Figure 6. Expression of cytokines in PPARG knockout LC cells. (A-D) Validation of the knockdown efficiency in (A, B) TU212 and (C, D) TU686 cell lines by western blot and quantified with Image J. (E-L) mRNA expression levels of cytokines quantified with qRT-PCR in control and PPARG knockout cells, including (E) CCL2, (F) CSF1, (G) PDGFC, (H) TGFB3, (I) FGF2, (J) IL1A, (K) IL18 and (L) CXCL11. * $p < 0.05$; ** $p < 0.01$; *** $p < 0.001$.

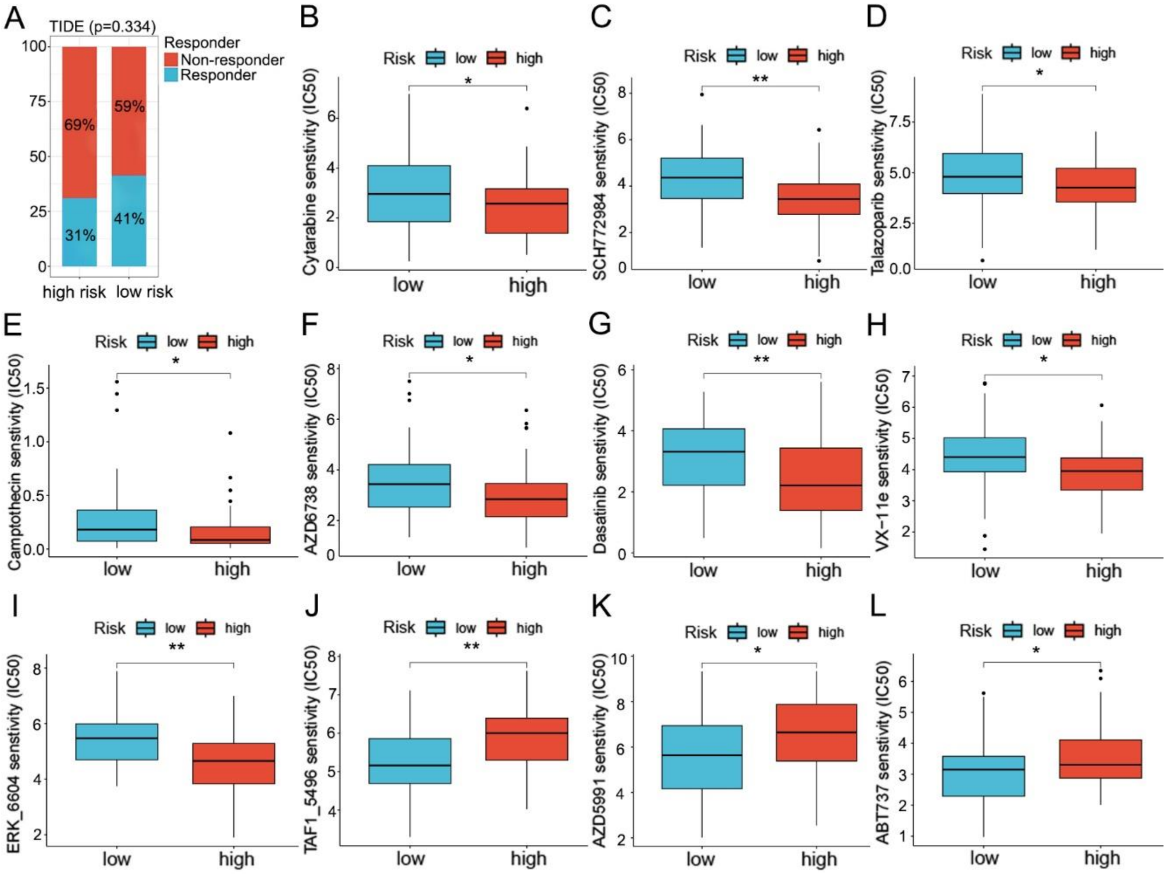

Supplementary Figure 7. Prediction of sensitivity to clinical therapeutic agents. (A) Predicted response to immunotherapy in the high- and low-risk groups based on the TIDE algorithm. (B-K) Predicted IC50 values for chemotherapy and targeted drugs, including (B) SCH772984, (C) talazoparib, (D) camptothecin, (E) AZD6738, (F) dasatinib, (G) VX-11e, (H) ERK-6604, (I) TAF1-5496, (J) AZD5991and (K) ABT737. Statistical significance was indicated between the high- and low-risk groups. * $p < 0.05$; ** $p < 0.01$.

Supplementary Table 1. The weblinks of databases and online tools used in this study.

Platform	Link
TCGA	https://portal.gdc.cancer.gov/
GEO	https://www.ncbi.nlm.nih.gov/geo/
iUUCD 2.0	http://iuucd.biocuckoo.org/
UbiBrowser 2.0	http://ubibrowser.ncpsb.org.cn
Sangerbox 3.0	http://sangerbox.com/
STRING	https://string-db.org/
SRplot	http://www.bioinformatics.com.cn/
GSEA	https://www.gsea-msigdb.org/gsea/index.jsp
Chiplot	https://www.chiplot.online/
GDSC	https://www.cancerrxgene.org/

Supplementary Table 2. Patient number and statistical significance of survival analysis.

Clinical Condition (Total Number)	Subgroups		*p*-Value
	High-risk	Low-risk	
Training set (n=116)	58	58	< 0.001
<60 years old (n=67)	33	34	< 0.001
>60 years old (n=38)	17	21	0.025
Male (n=86)	42	44	< 0.001
Female (n=19)	8	11	0.087
Grade1-2 (n=73)	41	32	<0.01
Grade3-4 (n=32)	9	23	0.043
T1-2 stage (n=19)	9	10	0.083
T3-4 stage (n=86)	41	45	< 0.001
N0-1 stage (n=69)	30	39	<0.01
N2-3 stage (n=36)	20	16	0.029
M0 stage (n=102)	48	54	< 0.001
M1 stage (n=3)	2	1	0.16
Clinical stage I-II (n=12)	7	5	0.13
Clinical stage III-IV (n=93)	43	50	< 0.001
PPARG (n=116)	81	35	<0.01
LCK (n=116)	60	56	0.012
LHX1 (n=116)	34	82	<0.01
Validation set (n=46)	24	22	0.016

Supplementary Table 3. sgRNA sequences for control and PPARG knockout.

Genes	Primer sequence (5’-3’)
PPARG	caccgACAGATGTGATCTTAACTGT
	aaacACAGTTAAGATCACATCTGTc
Control	caccgGTACCATACCGCGTACCCTT
	aaacAAGGGTACGCGGTATGGTACc

Supplementary Table 4. Primer sequences used for qRT-PCR.

Genes	Primer sequence (5'-3')
IL1A	TCAAGGAGAGCATGGTGGTAGTAG
	TCCTCTGAGTCATTGGCGATGG
IL6	GTGTTGCCTGCTGCCTTCC
	TCTGAAGAGGTGAGTGGCTGTC
IL18	CTGGAATCAGATTACTTTGGCAAGC
	TCCTTGGTCAATGAAGAGAACTTGG
CXCL11	TGCTACAGTTGTTCAAGGCTTCC
	ACTTGGGTACATTATGGAGGCTTTC
CCL2	CCAGCAGCAAGTGTCCCAAAG
	TGCTTGTCCAGGTGGTCCATG
VEGFC	AGTTACGGTCTGTGTCCAGTGTAG
	TGCCAGCCTCCTTTCCTTAGC
TGFB1	GCAACAATTCCTGGCGATACCTC
	CCTCCACGGCTCAACCACTG
TGFB2	GAGTGCCTGAACAACGGATTGAG
	GCCATTCGCCTTCTGCTCTTG
TGFB3	CTGTGCGTGAGTGGCTGTTG
	CCATTGGGCTGAAAGGTGTGAC
CSF1	GCCAGTGAGATTCCCGTACCC
	GGAGTGGAGAAGATGCTGAGAGG
FGF2	GGAGAAGAGCGACCCTCACATC
	CTTCATAGCCAGGTAACGGTTAGC
PDGFC	TTCGGGCTTCTCCTGCTGAC
	TTGTACTCCGTTCTGTTCCTTGTTG
GAPDH	ACACCCACTCCTCCACCTTTG
	TCCACCACCCTGTTGCTGTAG
